# USF1-ATRAP-PBX3 Axis Promote Breast Cancer Glycolysis and Malignant Phenotype by Activating AKT/mTOR Signaling

**DOI:** 10.7150/ijbs.69134

**Published:** 2022-03-14

**Authors:** Dandan Wang, Xiaoying Jin, Mengxia Lei, Ying Jiang, Yali Liu, Fei Yu, Yan Guo, Bing Han, Yue Yang, Weiling Sun, Ying Liu, Guangchun Zeng, Feng Liu, Jing Hu, Xuesong Chen

**Affiliations:** 1 Department of Breast Medical Oncology, Harbin Medical University Cancer Hospital, 150 Haping Road, Harbin 150040, China; 2 Department of Biochemistry and Molecular Biology, Mudanjiang Medical University, 3 Tongxiang Street, Mudanjang 157011, China; 3 Heilongjiang Academy of Medical Sciences, Harbin Medical University, 157 Baojian Road, Harbin 150081, China; 4 The Institute of Cancer Prevention and Treatment, Harbin Medical University Cancer Hospital, 150 Haping Road, Harbin 150040, China; 5 Department of Medical Oncology, Harbin Medical University Cancer Hospital, 150 Haping Road, Harbin 150040, China; 6 Department of Pathology, Harbin Medical University Cancer Hospital, 150 Haping Road, Harbin 150040, China; 7 Department of Breast Surgery, Harbin Medical University Cancer Hospital, 150 Haping Road, Harbin 150040, China

**Keywords:** Breast cancer, ATRAP, Glycolytic metabolism, AKT/mTOR signaling, Ubiquitination

## Abstract

Angiotensin II type 1 receptor-associated protein (ATRAP) is widely expressed in different tissues and organs, although its mechanistic role in breast cancer remains unclear. Here, we show that ATRAP is highly expressed in breast cancer tissues. Its aberrant upregulation promotes breast cancer aggressiveness and is positively correlated with poor prognosis. Functional assays revealed that ATRAP participates in promoting cell growth, metastasis, and aerobic glycolysis, while microarray analysis showed that ATRAP can activate the AKT/mTOR signaling pathway in cancer progression. In addition, ATRAP was revealed to direct Ubiquitin-specific protease 14 (USP14)-mediated deubiquitination and stabilization of Pre-B cell leukemia homeobox 3 (PBX3). Importantly, ATRAP is a direct target of Upstream stimulatory factor 1 (USF1), and that ATRAP overexpression reverses the inhibitory effects of USF1 knockdown. Our study demonstrates the broad contribution of the USF1/ATRAP/PBX3 axis to breast cancer progression and provides a strong potential therapeutic target.

## Introduction

Female breast cancer is the most common malignancy (accounting for 11.7% of all cancer cases), with an estimated 2.3 million new cases, globally, in 2020 1. In the USA, it is the second leading cause of cancer-related death, with an estimated 281,550 new cases and 43,600 deaths in 2021 2. Currently, treatments for breast cancer include surgery, chemotherapy, radiation therapy, and targeted therapy. Despite this array of treatments, the overall survival rate of breast cancer patients is still very low 3,4. Therefore, further exploration of the molecular mechanisms driving breast cancer progression is essential for developing effective treatment strategies to prevent breast cancer recurrence and metastasis and to improve overall survival outcomes.

Angiotensin II type 1 receptor-associated protein (ATRAP, also known as AGTRAP), a low molecular weight transmembrane protein encoded by 483 nucleotides, was originally identified by yeast two-hybrid screen 5. It contains three transmembrane domains at the amino-terminus that can interact with the C-terminal domain of the AT1 receptor and a hydrophilic cytoplasmic C-terminal tail 6. ATRAP is expressed in various tissues, including the aorta, heart, lung, vascular smooth muscle cells, and adipose tissues, but most strongly in renal tubules of the kidneys where it has been shown to co-localize and interact with the AT1 receptor in mice 7-10. Additionally, ATRAP was shown to potentially prevent tissue metabolic abnormalities, including lipid deposition and hepatic fibrosis 11, 12. Despite reported associations with kidney, liver, and heart disease, no evidence has thus far identified a relationship between ATRAP expression and tumor development in these tissues, nor any potential role of ATRAP in breast cancer.

The mammalian target of rapamycin (mTOR) is encoded by the mTOR gene, a 289 kDa serine/threonine protein kinase in the phosphatidylinositol-3-kinase (PI3K) family-related protein kinases (PIKKs) 13-15, which contains two different catalytic subunit protein complexes, including mTOR complex 1 (mTORC1) and mTOR complex 2 (mTORC2). AKT can mediated Mel18 phosphorylation regulates chromatin ubiquitylation modification and promotes tumor malignancy 16. The mTOR signaling pathway is activated by AKT and associated with several fundamental cellular processes 17, such as autophagy 18, and cancer 19. Abnormal mTOR activation can regulate several essential features of tumor formation including aberrant cellular metabolism, cell migration and invasion, and angiogenesis 20. This pathway is thus regarded as an attractive candidate for targeted molecular therapies.

In a phenomenon called the Warburg effect or aerobic glycolysis 21, 22, cancer cells exhibit a high level of glycolytic metabolism even in the presence of abundant oxygen, which in turn promotes tumor growth by increasing glucose uptake and lactic acid production. In addition, cancer cells are challenged by various environmental and cellular pressures during metastasis 23. In response, cancer cells can manipulate one or more metabolic pathways depending on their stage in the metastatic cascade and the sites of metastasis 24-27. However, the regulatory mechanisms that induce cancer proliferation and metastasis by metabolic reprogramming in breast cancer have not yet been characterized.

Here, in this work, we show that ATRAP is strongly upregulated in breast cancer and is significantly associated with prognosis in breast cancer patients. We discovered that elevated ATRAP transcription is mainly regulated by USF1 interaction with an ATRAP promoter, whereas ATRAP directs USP14-mediated deubiquitination and stabilization of PBX3. In addition, ATRAP can function as an oncogene through activation of the AKT/mTOR pathway in breast cancer. This study thus enhances our understanding of the metabolic and regulatory mechanisms that drive breast cancer progression, providing valuable insights necessary for development of novel therapeutic approaches for treatment of metastatic breast cancer.

## Materials and Methods

### Cell lines and culture conditions

The human breast cancer cell lines UACC-812, SKBR3, MDA-MB-453, T47D, MCF7, MDA-MB-468, and MDA-MB-231 were obtained from Heilongjiang Cancer Institute (Harbin, China). The UACC-812 and T47D cell lines were maintained in DMEM (Gibco, Life Technologies, California, USA), MDA-MB-453 and SKBR3 in RPMI-1640 (Gibco, Life Technologies, California, USA), The MDA-MB-468 and MDA-MB-231 cell lines in Leibovitz's L15 (Boster, Wuhan, China), all them were supplemented with 10% FBS (ScienCell, California, USA). The MCF-7 cell line was maintained in DMEM supplemented with 10% FBS and 0.01 mg/mL human recombinant insulin. For mTOR inhibitor assays, cells were treated with 20 nM rapamycin (MCE, New Jersey, USA) for 24 hours for functional verification and western blot analysis. For the ubiquitination assay, cells were treated with 10 μM MG132 (MCE, New Jersey, USA) for 6 h and protein levels were detected by immunoblotting. For the CHX chase assay, cells were exposed to 200 μg/mL CHX (MCE, New Jersey, USA) and then harvested at different times (0, 2, 4, 6, 8, and/or 10 h) for western blotting to detect the proteins of interest.

### Patient information and tissue specimens

Ten pairs of fresh frozen tumor and matched peritumor samples were randomly collected from the Harbin Medical University Cancer Hospital (Harbin, China) and analyzed by western blotting. We obtained paraffin-embedded samples from 159 patients (87 invasive ductal carcinoma tissues, 48 ductal carcinoma *in situ* tissues and 24 normal breast tissues) between March 2010 and March 2015 at the Harbin Medical University Cancer Hospital. Among them, 49 breast cancer tissue samples were randomly selected to analyze the correlation of ATRAP, USF1 and PBX3. A total of 362 paraffin-embedded breast cancer and 28 normal breast tissues were collected to construct tissue microarrays. All patients were subjected to a complete excision followed by tissue verification through pathological examination. The clinical and prognosis data were collected from 1 January 2007 to 31 August 2021. Prior to surgery, no patients had received any form of radiotherapy or chemotherapy, and detailed clinicopathological and follow-up data had been obtained. The use of human tissue was approved by the Institutional Research Ethics Committee of the Harbin Medical University Cancer Hospital, and informed consent was obtained from all patients.

### Vectors, lentivirus infection, and transfection

Knockdown (Vector, shATRAP) and overexpressed (Vector, ATRAP) lentivirus were constructed by Genechem (www.genechem.com.cn; Shanghai, China). For the overexpressed lentivirus, concentrated viruses were used to infect 5×10^5^ cells in complete medium containing 100 μl 1×HitransG P in six-well plates. For the knockdown lentivirus, concentrated viruses were used to infect 5×10^5^ cells in ENi.S containing 5 μg/mL Polybrene (www.genechem.com.cn; Shanghai, China) in six-well plates. Then, the infected cells were subjected to selection with 2 μg/mL puromycin (Clontech, Mountain View, CA, USA) for 72 h. Stable overexpression or knockdown cell lines were identified using qRT-PCR or western blot. Small interfering RNAs (siRNAs) against human PBX3, USP14, and USF1 were purchased from RiboBio Co., Ltd (Guangzhou, China), and non-specific siRNA was used as a negative control. Flag-ATRAP plasmid (Flag-ATRAP), PBX3 plasmid (PBX3), and USF1 plasmid (USF1) were subcloned into the expression vector pCMV3 (Sino Biological Inc., Beijing, China), while empty vector plasmid was used as a negative control. All of the siRNAs and plasmids were transfected using jetPRIME^®^ (Polyplus-transfection S.A, Illkirch, France) according to the manufacturer's instructions. The target sequences are provided in Table S1.

### Cell viability

For the CCK-8 assay, cells were seeded in 96-well plates. At various time points (1, 2, 3, 4, 5 days and/or 12, 24, 48, 60 hours), groups of cells were incubated with 10 μl Cell Counting Kit-8 (Dojindo Laboratories, Kumamoto, Japan) for 2 h at 37°C. The absorbance values were measured at 490 nm. For the colony formation assay, cells were inoculated in a six-well plate, incubated at 37°C, 5% CO_2_, and fixed for 1 h after 14 days. Crystal violet was added for staining overnight, then each well was washed three times and counted.

### Transwell and wound healing assays

For transwell assays, a certain number of cells (8✕10^4^ cells per well for UACC-812 and T47D, 10^5^ cells per well for MDA-MB-453) were suspended in 200 μl of serum-free medium and placed in the top inserts with or without Matrigel (BD Biosciences, San Jose, CA, USA). After 24-48 h (migration is 24 h; invasion is 48 h), the cells in the bottom surface were fixed and stained with crystal violet. Five randomly selected visual fields on each insert were photographed, and cells were counted manually. For wound healing assays, artificial wounds were scratched on a confluent cell monolayer using 10 μl pipette tips until cells covered 95% of the 6-well plate bottom. Then wound healing images were taken at 0 h and 24/48 h (UACC-812 is 24 h; T47D and MDA-MB-453 are 48 h).

### Quantitative reverse transcriptase PCR

Total RNA was extracted with TRNzol reagent (TIANGEN Biotech, Beijing, China) and used to generate cDNA by FastKing one-step removal of genomic cDNA first-strand synthesis premix reagent (TIANGEN Biotech, Beijing, China) and cDNA synthesis for SuperReal PreMix Plus (TIANGEN Biotech, Beijing, China) with an oligo-dT primer. Real-time reverse transcriptase PCR (qRT-PCR) was performed using SYBR Select Master Mix (Life Technologies) as recommended by the manufacturer. GAPDH was used as the internal control. Primers were as follows:

5′-CCACCATCTTCCTGGACATC-3′ (forward) and 5′-TGAGTCAATCGTCTGGTAGGC-3′ (reverse) for ATRAP;

5′-CCAGACGGAAAAGGCGTAAC-3′ (forward) and 5′- ATTTCTTGGCCAGCTCCTCTT-3′ (reverse) for PBX3;

5′-TGACTTCAACAGCGACACCCA-3′ (forward) and 5′-CACCCTGTTGCTGTAGCCAAA-3′ (reverse) for GAPDH.

### Western blotting analysis

Western blotting was performed as previously described 28. Briefly, cells were lysed on ice in RIPA buffer, and protein concentrations were determined. Equal amounts of protein were subjected to electrophoresis on SDS-PAGE gels followed by immunoblot assays with the antibodies listed in Table S2.

### *In vivo* assay

BALB/c-nu mice (4-5 weeks of age, 18-20 g) were purchased from Vital River (Beijing, China). All experimental procedures were approved by the Ethics Committee of the Institutional Animal Care and Use Committees of Harbin Medical University. UACC-812 cells (6×10^6^ in 0.2 ml PBS/Matrigel [3:1]) transduced with either vector or shATRAP were injected into armpit regions (n=6/group). Approximately 6×10^6^ T47D cells transduced with either vector or ATRAP and a luciferase reporter in 0.2 ml of PBS/Matrigel [3:1] were separately injected into the armpit regions. After formation of a palpable tumor, the mice carrying T47D/ATRAP cells were randomly divided into two subgroups (n=5/group). Rapamycin (2 mg/kg) was delivered by intraperitoneal injection every other day to the appropriate groups for a total of 32 days. Tumor sizes were measured in perpendicular dimensions using vernier calipers every 8 or 9 days. Volumes were estimated using the formula (L*W^2^)/2, where L is the longer and W is the shorter of the 2 dimensions. Subsequently, 100 mg/kg D-luciferin was injected intraperitoneally into the groups of T47D cell mice, and tumor size was monitored by measuring the bioluminescence signal every week until all mice were sacrificed for collecting tumor tissues after three weeks of treatment. The *p*-value was obtained by comparisons between the control and treatment groups at each time point.

### Measurement of glucose and lactate

The concentrations of glucose and lactate in the cells of ATRAP treatment and control groups were determined by spectrophotometric analysis using assay kits (Glucose Assay Kit and Lactate Assay Kit, Nanjing Jiancheng Bioengineering Institute, Nanjing, China). All measurements followed manufacturer's instructions and were normalized for the number of cells in each experiment.

### Immunohistochemistry (IHC)

Paraffin-embedded tissue blocks were used for immunohistochemical staining. Paraffin-embedded tissue specimens were sectioned, deparaffinized in xylene, and rehydrated, followed by antigen retrieval in sodium citrate or EDTA. The sections were incubated with primary antibodies overnight at 4°C, and then incubated with horseradish peroxidase (HRP)-labeled secondary antibody (Beyotime, Shanghai, China). Specimens were stained using a DAB kit (Beyotime, Shanghai, China) until the desired stain intensity developed. Sections were then counterstained with hematoxylin, dehydrated, and covered by coverslip. The scores were determined by combining the proportion of positively-stained tumor cells and the intensity of staining. The proportion of positively stained tumor cells in a field were scored as follows: 0, no positive tumor cells; 1, <10% positive tumor cells; 2, 10-35% positive tumor cells; 3, 35-75% positive tumor cells; 4, ≥75% positive tumor cells. Staining intensity was graded according to the following standard: 1, no staining; 2, weak staining (light yellow); 3, moderate staining (yellow brown); 4, strong staining (brown). The staining index (SI) for each sample was obtained by multiplying the intensity and proportion values, and SI ≥ 8 was determined as high expression and samples with SI < 8 were determined to have low expression.

The protein expression levels of ATRAP, PBX3, and USF1 in human tumor tissues were assessed by IHC with the corresponding anti-ATRAP (dilution 1:100, ab85175, Abcam, Cambridge, USA), anti-PBX3 (dilution 1:50, 12571-1-AP, Proteintech, Wuhan, China), and anti-USF1 antibodies (dilution 1:200, A13560, ABclonal, Wuhan, China), respectively. Subsequently, the indicated protein expression levels were assessed by IHC with the corresponding anti-ATRAP (dilution 1:100, 11559-1-AP, Proteintech, Wuhan, China), anti-PBX3 (dilution 1:40, 12571-1-AP, Proteintech, Wuhan, China), anti-Ki67 antibodies (dilution 1:200, 27309-1-AP, Proteintech, Wuhan, China), anti-p-mTOR antibodies (dilution 1:100, AF3308, Affinity Biosciences, USA), and anti-E-cadherin antibodies (dilution 1:200, 20874-1-AP, Proteintech, Wuhan, China) in mice tumor tissues. The tissue microarray was evaluated through IHC staining of human tumor tissues with the anti-ATRAP antibody (dilution 1:200, 11559-1-AP, Proteintech, Wuhan, China).

### Immunofluorescence

The UACC-812 cells transfected with Flag-ATRAP plasmid were fixed with 4% formaldehyde, permeabilized with PBS containing 0.3% Triton X-100 (Beyotime, Shanghai, China), and blocked with 10% BSA. The cells were incubated with the indicated primary antibodies against Flag (dilution 1:25, 20543-1-AP, Proteintech, Wuhan, China) overnight at 4°C and then with DyLight 488-conjugated anti-mouse IgG (1:200 dilution, A23210, Abbkine, Wuhan, China) in the dark at room temperature for 1h. The stained cells were observed and captured using a laser-scanning confocal microscope.

### Silver staining

Silver staining was performed according to the protocol provided by Beyotime Technology (Beyotime, Shanghai, China). Briefly, the gels were fixed in fixative (50% ethanol, 10% acetic acid and 40% Milli-Q water) for 40 min after electrophoresis, wash in 30% ethanol and Milli-Q water for 10 minutes. The gels were incubated with silver staining sensitizer for 2 min and Milli-Q water wash twice, and then with silver nitrate for 10 min. Afterwards, the gels were put in Milli-Q water for 1 min, removed, and in the developing solution for 3-10 min. When clear staining was achieved, the gels were transferred to stop solution for 10 min. The stained gels were stored in Milli-Q water at 4°C.

### Immunoprecipitation (IP) assay

Co-immunoprecipitation (co-IP) assay was performed with the Pierce™ Crosslinked Magnetic Bead IP/Co-IP Kit (Thermo, Waltham, USA). The measurements were performed based on the instructions provided by the manufacturer. Briefly, the magnetic beads were pre-washed twice with 1×modified cross-linking buffer and the antibody (5 μg) was allowed to bind to the magnetic beads for 15 minutes, followed by washing the magnetic beads three times with 1×modified cross-linking buffer. The antibody and magnetic beads were cross-linked with DSS for 30 minutes, then rinsed three times with eluent, and finally washed twice with immunoprecipitation lysate. Next, the cell lysate was combined with the antibody crosslinked beads and held overnight at 4ºC. Finally, the bound antigen was eluted and subjected to western blot analysis.

### Ubiquitination assay

Cells were incubated in culture media and total proteins were extracted and co-IP was performed using anti-PBX3. The protein complexes were then subjected to western blot using anti-ubiquitin antibody, anti-PBX3 antibody and anti-ATRAP antibody to evaluate the proteasome-dependent ubiquitination level.

### Luciferase reporter assay

293T cells were cultured at a density of 2×10^4^ cells/well in 96-well culture plates and transfected with 0.2 μg of dual-luciferase reporter construct pcDNA3.1+ATRAP-WT and pcDNA3.1+ATRAP-Mut, or co-transfected with 0.2 μg of the luciferase reporter construct USF1+ATRAP-WT and USF1+ATRAP-Mut, and the internal control vector pRL-TK (Promega, Madison, WI) at a ratio of 20:1 (reporter construct: control vector) using Lipofectamine^TM^ 2000 (Invitrogen, Carlsbad, CA) according to the manufacturer instructions. Five hours post-transfection, the transfection medium was removed and replenished with medium containing 6 μM of curcumin (Sigma-Aldrich, St. Louis, MO) solubilized in 100% dimethylsulfoxide (DMSO) (Sigma). At 48 h post-transfection, luciferase activity was measured using the Dual-Luciferase^®^ Reporter Assay System (Promega). *Renilla* luciferase activity was normalized to firefly luciferase activity in cells transfected with the dual-luciferase reporter construct pcDNA3.1+ATRAP-WT and pcDNA3.1+ATRAP-Mut, and firefly luciferase activity was normalized to *Renilla* luciferase activity in cells co-transfected with the reporter construct USF1+ATRAP-WT and USF1+ATRAP-Mut and the control vector.

### Statistical analysis

Data are presented as the mean ± SEM of at least three independent experiments for each cellular experimental group and at least five independent experiments for each animal group. Student's *t*-tests and ANOVA were used to determine statistically significant differences between groups. Correlations between ATRAP expression and a pathological response were determined by the Chi-square or Fisher's test. The Overall Survival (OS) and Disease-Free Survival (DFS) were calculated as the time from surgery until the occurrence of death and relapse, respectively. Survival curves were plotted using the Kaplan-Meier method and compared by the log-rank test. The *p* < 0.05 indicated statistical significance. Detailed information is described in each figure legends. Statistical analysis was performed using SPSS 22.0 statistical software package and GraphPad Prism software (GraphPad Software Inc., San Diego, CA).

## Results

### ATRAP expression in breast cancer tissues is correlated with breast cancer progression and poor prognosis

**Figure 1:**
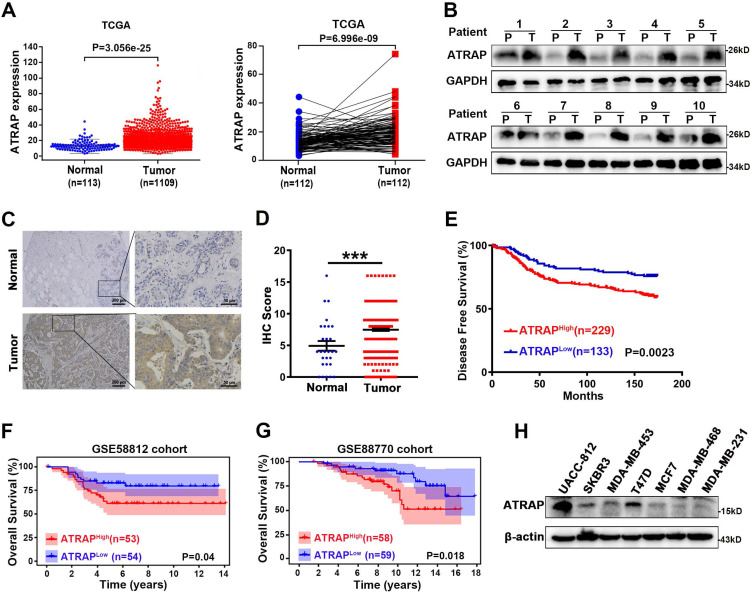
** Overexpression of ATRAP promotes breast cancer progression and is associated with poor prognosis in human breast cancer tissues. (A)** Expression profile of ATRAP mRNA in primary breast cancer tissues (n=1,109) and normal breast tissues (n=113). ATRAP was overexpressed in cancer compared with in 112 pairs of noncancerous adjacent tissues.** (B)** Expression of ATRAP, as determined by western blot, in ten paired primary breast cancer tissues (T) and the peritumor tissues (P). GAPDH served as a loading control.** (C)** Representative image of the IHC staining of ATRAP in a breast cancer tissue microarray. Scale bar, 200 μm and 50 μm. **(D)** The proportion of ATRAP expression levels in breast cancer and normal tissues. **(E)** Kaplan-Meier analyses of the relationships between ATRAP expression and DFS in breast cancer patients in the HMUCC cohort. **(F** and **G)** Kaplan-Meier analyses of the relationships between ATRAP expression and OS in breast cancer patients in the GSE58812 **(F)** and GSE88770 **(G)** cohorts. **(H)** Western blot analysis of ATRAP expression in seven breast cancer cell lines. β-actin served as a loading control. Data represent mean ± SEM. Statistical significance was determined by two-tailed unpaired *t*-test **(D)** or log-rank test **(E-G)**. ^*^p < 0.05, ^**^p < 0.01, ^***^p < 0.001, and ^****^p < 0.0001.

In order to better understand how Angiotensin II type 1 receptor-associated protein (ATRAP) contributes to breast cancer progression, we first examined publicly available expression profiles in The Cancer Genome Atlas (TCGA) (https://tcga-data.nci.nih.gov/). We found that the expression of ATRAP in primary breast cancer was significantly higher than that in normal breast tissues, as well in 112 pairs of cancer and noncancerous adjacent tissues (Figure 1A). Consistent with these findings, comparative analysis revealed that ATRAP was markedly overexpressed in 10 primary breast cancer samples compared with matched adjacent normal breast tissues (Figure 1B). We next analyzed its protein expression in breast tissue to determine its clinical significance using immunohistochemical (IHC) staining to examine a tissue array of 362 samples from breast cancer patients and 28 samples from normal breast tissues. Imaging revealed that ATRAP protein accumulated to significantly higher levels in breast cancer tissues compared with that in normal tissue (Figure 1C and D).

Furthermore, elevated ATRAP expression in breast cancer patients was correlated with a shorter disease-free survival (DFS) time compared to those of patients with low ATRAP expression in the HMUCC cohort (Figure 1E). Next, we downloaded the whole transcriptomes of two breast cancer studies (GSE58812 and GSE88770) from the GEO database (http://www.ncbi.nlm.nih.gov/geo/) to identify potential correlations between ATRAP expression and patient survival. And Kaplan-Meier survival analysis revealed that breast cancer patients with higher expression of ATRAP had shorter overall survival (Figure 1F and G). Analysis of the 362 breast cancer tissue array revealed that ATRAP protein levels were correlated with lymph node staging, but were not significantly correlated with age, tumor size, histological stage, pathological types, TNM stage, or other clinical pathological characteristics (ER, PR, HER2, Ki67 and P53) (Table 1). Importantly, univariate and multivariate analyses indicated that the ATRAP level, and node stage were independent indicators for breast cancer patient prognoses (Table 2). In addition, analysis of 159 breast tissue specimens showed that ATRAP expression increased gradually in normal breast tissue, ductal carcinoma *in situ* (DCIS) and invasive ductal carcinoma (IDC) (Figure S1A). The positive expression rates of ATRAP were 8.33% (2/24), 66.67% (32/48) and 73.56% (64/87), respectively (Figure S1B). Subsequently, we used western blotting to generate protein expression profiles for ATRAP in a panel of seven human breast cancer cell lines (Figure 1H), which showed relatively high expression in UACC-812, moderate expression levels in T47D; and low ATRAP protein in MDA-MB-453 cells. Thus, MDA-MB-453 and T47D expressing empty vector vs. ATRAP using lentivirus expression system and overexpression plasmid, respectively; Whereas UACC-812 and T47D stably expressing empty vector vs. ATRAP knockdown using lentivirus expression system were established to explore molecular mechanisms. Besides, we detected the cellular localization of ATRAP in UACC-812 cells by immunofluorescence assay (Figure S1C). The results showed that ATRAP was expressed in cytoplasm. Collectively, these results suggested that ATRAP expression was elevated and associated with poor prognosis in breast cancer tissues.

To explore the biological role of ATRAP down-regulation in breast cancer progression, we established lentiviral RNAi UACC-812 and T47D cell lines that stably suppressed ATRAP (Figure S2A). We observed that the ability of these breast cancer cell lines to proliferate under ATRAP suppression was weakened compared to control cells (Figure S2B). In addition, we found similar patterns of decreased long-term colony formation in these RNAi lines compared to control cells (Figure S2C). Moreover, transwell and wound-healing assays further showed that ATRAP suppression in both cancer cell lines resulted in markedly impaired capabilities of migration and invasion (Figure S2D and E). Consistent with these results, western blot analysis showed that ATRAP silencing effectively increased E-cadherin protein level, but decreased the accumulation of mesenchymal markers (N-cadherin and vimentin) in both UACC-812 and T47D cells (Figure S2F). Based on these findings, we concluded that suppression of ATRAP inhibits breast cancer aggressiveness *in vitro*.

In light of these findings showing the effects of decreased ATRAP on cancer cells, we next investigated the effects of ATRAP overexpression in these cell lines. To this end, we generated MDA-MB-453 ATRAP stable-overexpression lines and empty vector controls using a lentiviral expression system, as well as a T47D cell line carrying the pCMV3-Flag-ATRAP vector construct and a corresponding empty vector control line. Western blot and PCR analysis showed that the transfection efficiency was suitable for further experiments (Figure S3A). CCK-8 and colony formation assays then indicated that overexpression of ATRAP resulted in a significant increase in cell proliferation in these overexpression cell lines compared with controls (Figure S3B and C). Moreover, transwell and wound-healing assays showed that ATRAP-overexpression in these breast cancer cells promoted their ability to metastasize (Figure S3D and E). Consistent with these findings, ATRAP overexpression led to reduced E-cadherin protein levels, but elevated N‑cadherin and Vimentin (Figure S3F).

### ATRAP activates AKT/mTOR signal pathway and enhances glycolytic metabolism

**Figure 2:**
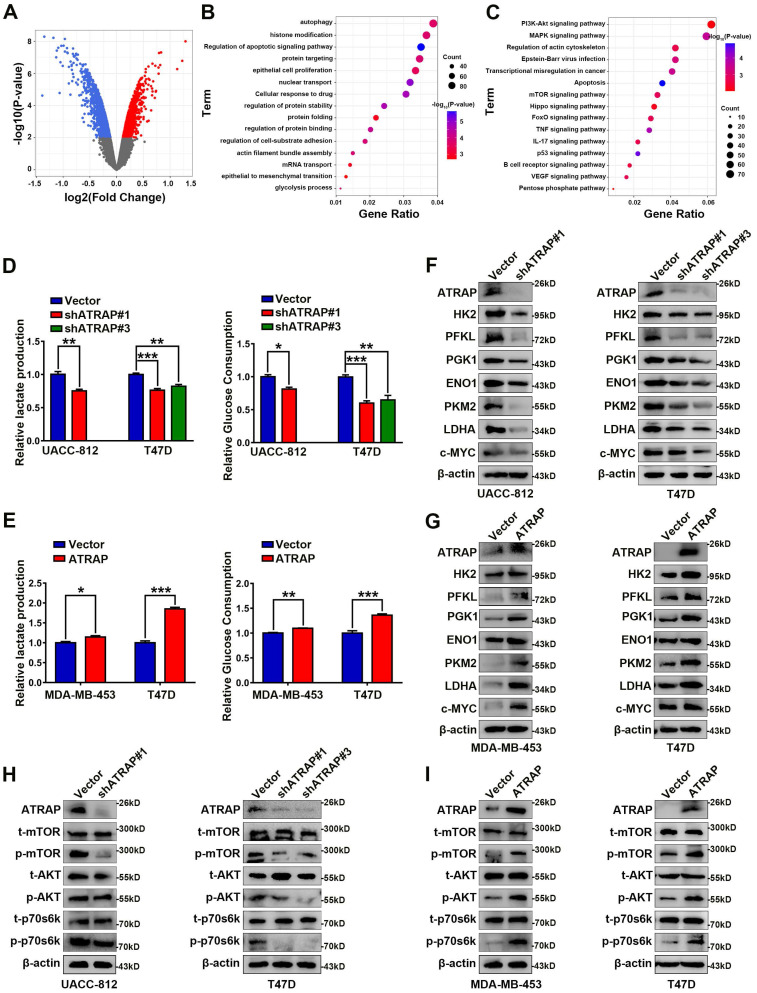
** ATRAP facilitates metabolic rewiring reprogramming of breast cancer cells and activates AKT/mTOR signaling. (A)** A volcano plot of the differentially expressed genes (DEGs). Globally differentially expressed genes (red means up-regulated and blue means down-regulated) are defined as *p-*value < 0.05. **(B**, **C)** GO functional enrichment analysis (**B**) and KEGG pathway enrichment analysis (**C**) (indicated by the inverse log10 of *p* values). **(D)** Relative lactate production level and relative glucose consumption level was determined in UACC-812 and T47D cells transfected with empty vector or ATRAP-shRNA. **(E)** Relative lactate production level and relative glucose consumption level were determined in ATRAP-overexpressing cells. **(F, G)** Western blot analysis of aerobic glycolysis enzymes and c-MYC in ATRAP-knockdown cells and overexpressing cells. **(H, I)** Western blot analyses of the expression levels of the indicated proteins in ATRAP knockdown and overexpressing cells. β-actin served as a loading control. Data represent mean ± SEM. Statistical significance was determined by two-tailed unpaired *t*-test (**D, E**). ^*^p < 0.05, ^**^p < 0.01, and ^***^p < 0.001.

To explore the potential mechanism by which ATRAP influences breast cancer progression, we performed microarray analysis in UACC-812 cells which ATRAP was depleted, to examine the differentially expressed genes (DEGs) of breast cancer cells (*p* < 0.05) (Figure 2A). GO functional enrichment analyses suggested that ATRAP participates in a variety of cellular functions, including cell substrate adhesion, apoptosis, autophagy, and glycolysis (Figure 2B). In addition, we found that ATRAP was enriched in multiple breast cancer-related signaling pathways by KEGG enrichment analyses, such as the P53 signaling pathway, the mTOR pathway, and apoptosis-related pathways (Figure 2C). We subsequently identified that decreased activation of mTOR signaling (notable downregulation of the DEGs in the mTOR pathway) was observed in ATRAP knockdown cells compared with the control cells. (Figure S4, Table S3).

Based on these results, we next examined differences in metabolism related to ATRAP expression, since cancer cells are known to exhibit abnormal metabolism, even under adequate oxygen, and it is widely accepted that aerobic glycolysis is essential for cancer proliferation and metastasis. Moreover, tumor progression may activate several metabolism-related signaling pathways, consequently leading to metabolic reprogramming 29. In our study, we found that knockdown of ATRAP reduced glucose consumption and lactate production in UACC-812 and T47D cells (Figure 2D). Conversely, ATRAP overexpression substantially enhanced glucose consumption and lactate production in MDA-MB-453 and T47D cells (Figure 2E). In addition, the expression of glycolysis-related enzymes (including HK2, PFKL, PGK1, ENO1, PKM2, LDHA and c-MYC) were remarkably decreased in shATRAP cells (Figure 2F), whereas these genes were all up-regulated in ATRAP overexpression cell lines (Figure 2G). Subsequent western blot analysis showed that expression of mTOR signaling pathway protein phosphorylation level was down-regulated in ATRAP-silenced cells (Figure 2H), but increased in ATRAP-overexpressing cells (Figure 2I). Taken together, these findings indicated that ATRAP promotes AKT/mTOR signaling and enhances glycolytic metabolism.

### ATRAP functions as an oncogene via AKT/mTOR signaling

**Figure 3:**
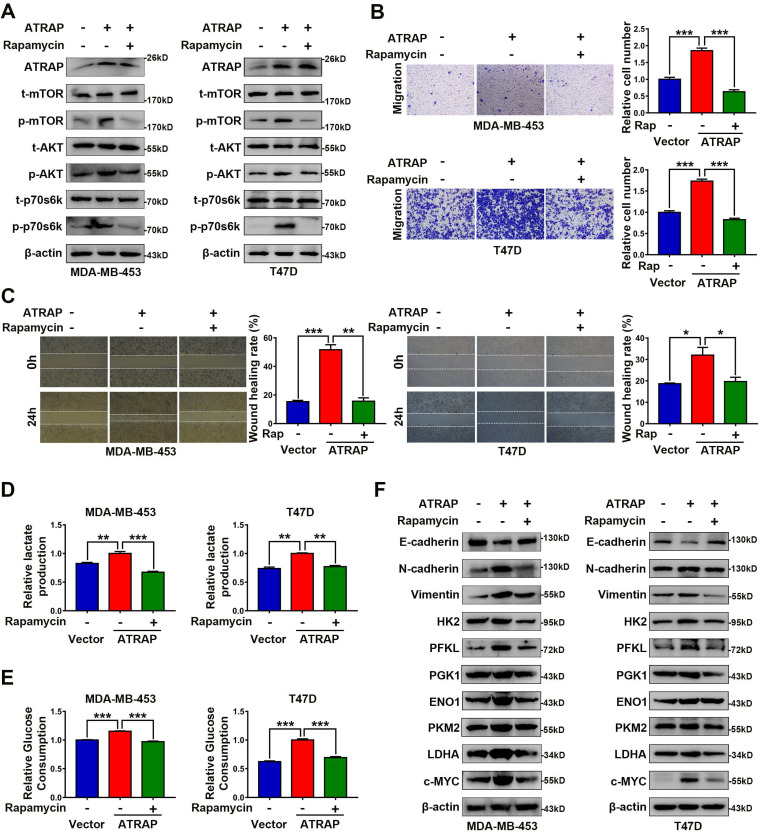
** AKT/mTOR signaling is essential for ATRAP-mediated EMT and glycolytic metabolism. (A)** Western blot analysis of key signal transduction proteins in vector control and ATRAP-overexpressing cells with or without rapamycin (mTOR inhibitors, 20 nM). **(B)** MDA-MB-453/T47D-overexpressig cells seeded in transwell culture chambers were treated with rapamycin. After 24 h, the migratory cells on the lower surface of the filter were stained with crystal violet and counted. **(C)** A wound healing assay was used to examine changes in the migration ability of ATRAP-overexpression cells cultured with rapamycin. **(D, E)** Relative lactate production level and relative glucose consumption level were determined in ATRAP-overexpressing cells with or without rapamycin. **(F)** Protein expression levels of E-cadherin, N-cadherin, vimentin, HK2, PFKL, PGK1, ENO1, PKM2, LDHA, and c-MYC were analyzed by western blotting. β-actin served as a loading control. Data represent mean ± SEM. Statistical significance was determined by two-tailed unpaired *t*-test (**B-E**). ^*^p < 0.05, ^**^p < 0.01, and ^***^p < 0.001.

Based on these results showing an apparent relationship between AKT/mTOR signaling and ATRAP expression in breast cancer cells, we next used the mTOR inhibitor rapamycin (20 nM) to examine whether AKT/mTOR signaling was required for ATRAP-mediated breast cancer progression. Western blot analysis indicated that rapamycin treatment reversed the increase in the accumulation of activated (i.e., phosphorylated) AKT/mTOR proteins observed in ATRAP-overexpressing breast cancer cell lines (Figure 3A). Similarly, rapamycin treatment also eliminated the increase in migration and invasion by MDA-MB-453 and T47D cells overexpressing ATRAP that we previously observed in transwell and wound-healing assays (Figure 3B and C). Exposure to rapamycin also led to a significant decrease in glucose consumption and lactate production among ATRAP-overexpression cells compared with untreated cells (Figure 3D and E). In addition, we used western blot analysis to examine the protein expression of EMT markers and glycolysis-related enzymes under treatment with rapamycin and found that rapamycin treatment increased the expression of epithelial tissue marker (E-cadherin) but decreased the expression of mesenchymal markers (N-cadherin, Vimentin) and glycolysis-related kinases (HK2, PFKL, PGK1, ENO1, PKM2, LDHA and c-MYC) in MDA-MB-453 and T47D ATRAP-overexpression cell lines (Figure 3F). Overall, these data suggested that mTOR activation is critical for the effects of ATRAP in increasing metabolism and invasiveness of breast cancer cells.

### ATRAP guides USP14-mediated deubiquitination and stabilization of PBX3

**Figure 4:**
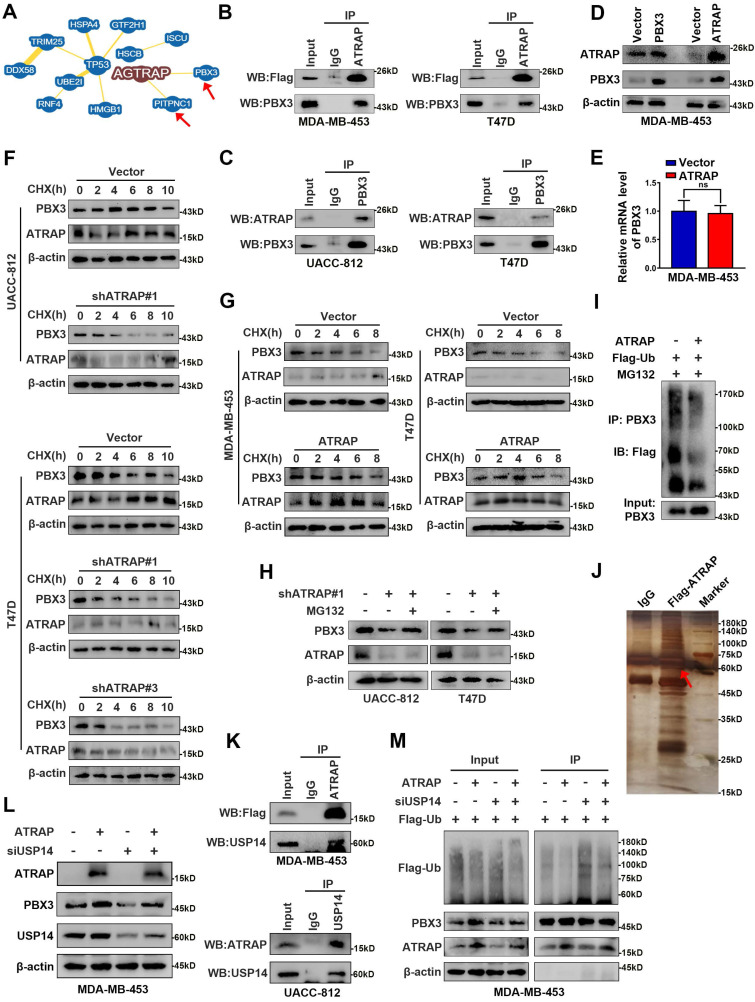
** ATRAP directs USP14-mediated de-ubiquitination and stabilization of PBX3. (A)** From BioGRID database (https://thebiogrid.org/), screen the data with the “MINIMUM EVIDENCE” of four showed the two proteins that directly interact with ATRAP. **(B)** IP analysis interaction between transfected Flag-ATRAP and endogenous PBX3 in MDA-MB-453 and T47D cells. **(C)** Interaction between endogenous ATRAP and PBX3 in UACC-812 and T47D cells. **(D)** Western blots were used to detect expression of ATRAP and PBX3 in indicated cells. **(E)** qRT-PCR was used to detect expression of PBX3 in indicated cells. **(F)** Immunoblots of cancer cells stably expressing vector control and shATRAP were treated with CHX (200 μg/mL) for the indicated time points. **(G)** Immunoblots of cancer cells treated with CHX for the indicated time points. **(H)** Immunoblots of UACC-812 and T47D cells expressing shATRAP#1 were treated with or without MG132 (10 μM) for 6 h. **(I)** Ubiquitination assay for the effects of ATRAP on PBX3 ubiquitination. Flag-Ub were co-transfected into MDA-MB-453/Vector and MDA-MB-453/ATRAP cells. **(J)** Flag-ATRAP vector was transfected into MDA-MB-453 cells, and anti-Flag was used to immunoprecipitate ATRAP-binding proteins. After silver staining, the ATRAP-specific bands were excised and analyzed by mass spectrometry (the arrow points to the location of the target protein). **(K)** Co-IP analysis of MDA-MB-453 and UACC-812 cells with an anti-Flag and anti-USP14 antibody, respectively, and analyzed by immunoblotting. **(L)** MDA-MB-453/Vector and MDA-MB-453/ATRAP cells transfected with USP14 siRNA. Western blot analysis the indicated cell lysates. **(M)** PBX3 and Flag-Ub were co-expressed with USP14-siRNA in MDA-MB-453/Vector or MDA-MB-453/ATRAP cells. PBX3 was immunoprecipitated and the polyubiquitination of PBX3 was detected by immunoblotting. β-actin served as a loading control. Data represent mean ± SEM. Statistical significance was determined by two-tailed unpaired *t*-test (**E**). ^*^p < 0.05, ^**^p < 0.01, and ^***^p < 0.001.

Indeed, while our results demonstrated that ATRAP regulated mTOR signaling, we did not observe a direct interaction between these proteins. Therefore, to resolve the underlying mechanism by which ATRAP promotes breast cancer progression, we used the BioGRID database (https://thebiogrid.org/) to explore potential ATRAP interaction partners, which revealed a variety of candidate proteins, among which we selected PBX3 for closer scrutiny based on its reported function in promoting tumor migration and invasion 30, 31 (Figure 4A and Table S4). To determine whether PBX3 participates in ATRAP-mediated breast cancer progression, we generated a PBX3 siRNA knockdown line in UACC-812 cells (Figure S5A). Similar effects to those with ATRAP knockdown were observed in PBX3 knockdown breast cancer cells. Specifically, PBX3 knockdown significantly inhibited the proliferation and migration of UACC-812 cells compared with controls (Figure S5B-D). Moreover, PBX3 suppression also interfered with AKT/mTOR pathway activation, and negatively affected the expression of EMT markers and glycolytic kinases (Figure S5E). To assess whether ATRAP and PBX3 proteins directly interact, we performed co-IP assays in MDA-MB-453 and T47D cells transfected with Flag-tagged ATRAP, which revealed that ATRAP can directly bind with PBX3 (Figure 4B). Furthermore, the interaction between endogenous PBX3 and ATRAP was also demonstrated by co-IP with an antibody against endogenous PBX3 in UACC-812 and T47D cells (Figure 4C). These results indicated that ATRAP specifically interacts with PBX3 in breast cancer cells.

Furthermore, we found that overexpression of ATRAP resulted in significant upregulation of PBX3 protein, whereas overexpression of PBX3 did not influence the expression of ATRAP (Figure 4D). These results suggested that PBX3 serves as substrate for ATRAP. In addition, ATRAP influenced PBX3 protein expression, but not its mRNA transcription (Figure 4E). We thus hypothesized that ATRAP could potentially regulate PBX3 protein stability. To explore this possibility, we examined the effects of both ATRAP depletion and overexpression on the stability of endogenous PBX3 protein in the presence of the protein synthesis inhibitor, cycloheximide (CHX). We found that the half-life of PBX3 protein was markedly reduced in ATRAP-knockdown cells compared to control cells (Figure 4F). Consistent with this observation, ATRAP overexpression in MDA-MB-453 and T47D cells increased the half-life of PBX3 protein in the presence of CHX (Figure 4G). Notably, we observed that the reduced PBX3 protein levels observed under ATRAP suppression in UACC-812 and T47D cells could be apparently recovered by the addition of proteasome inhibitor (MG132) (Figure 4H). Subsequent ubiquitination assays showed that overexpression of ATRAP resulted in decreased PBX3 protein polyubiquitination (Figure 4I). These results suggested that the ATRAP could potentially regulate PBX3 protein levels via inhibition of proteasomal degradation.

Next, to further validate the role of PBX3 in ATRAP biological function, we conducted complementation experiments. To this end, PBX3 plasmid was transfected into ATRAP-knockdown UACC-812 and T47D cell lines, which resulted in significant rescue of the decreased cell migration (Figure S6A and B), lactate production and glucose consumption (Figure S6C and D). In addition, PBX3 expression restored the levels of AKT/mTOR proteins that were decreased by ATRAP suppression, while also alleviating the inhibitory effects on EMT marker and glycolysis-related kinase protein levels in ATRAP knockdown cell lines (Figure S6E). Taken together, these results demonstrated that ATRAP regulates aggressiveness in breast cancer cells via PBX3.

Ubiquitination is a dynamic process involving ubiquitin ligases and deubiquitinases (DUBs). To elucidate the mechanism by which ATRAP interacts with and stabilizes PBX3 protein, we conducted co-immunoprecipitation assays followed by mass spectrometry to screen ATRAP-interacting proteins. Mass spectrometry data (Table S5) indicated that the USP14, one of three proteasome-related deubiquitinating enzymes that can remove ubiquitin from the proteasome substrate before it is degraded 32, was a strong candidate for further binding validation (Figure 4J). We subsequently performed co-IP assays with a Flag-tagged ATRAP or USP14 antibody to validate whether ATRAP interacts with endogenous USP14. The results showed that ATRAP indeed directly interacts with USP14 in MDA-MB-453 and UACC-812 cells (Figure 4K). Furthermore, as expected, western blot showed that depletion of USP14 by siRNA in MDA-MB-453 cells resulted in significantly reversed the ATRAP overexpression-induced increase in PBX3 (Figure 4L). We further verified that USP14 knockdown could reduce ATRAP induced PBX3 stabilization (Figure 4M). Cumulatively, these findings showed that ATRAP stabilizes PBX3 protein levels by blocking its proteasomal degradation, potentially through recruitment of the deubiquitinase USP14.

### ATRAP contributes to breast cancer progression *in vivo*

**Figure 5:**
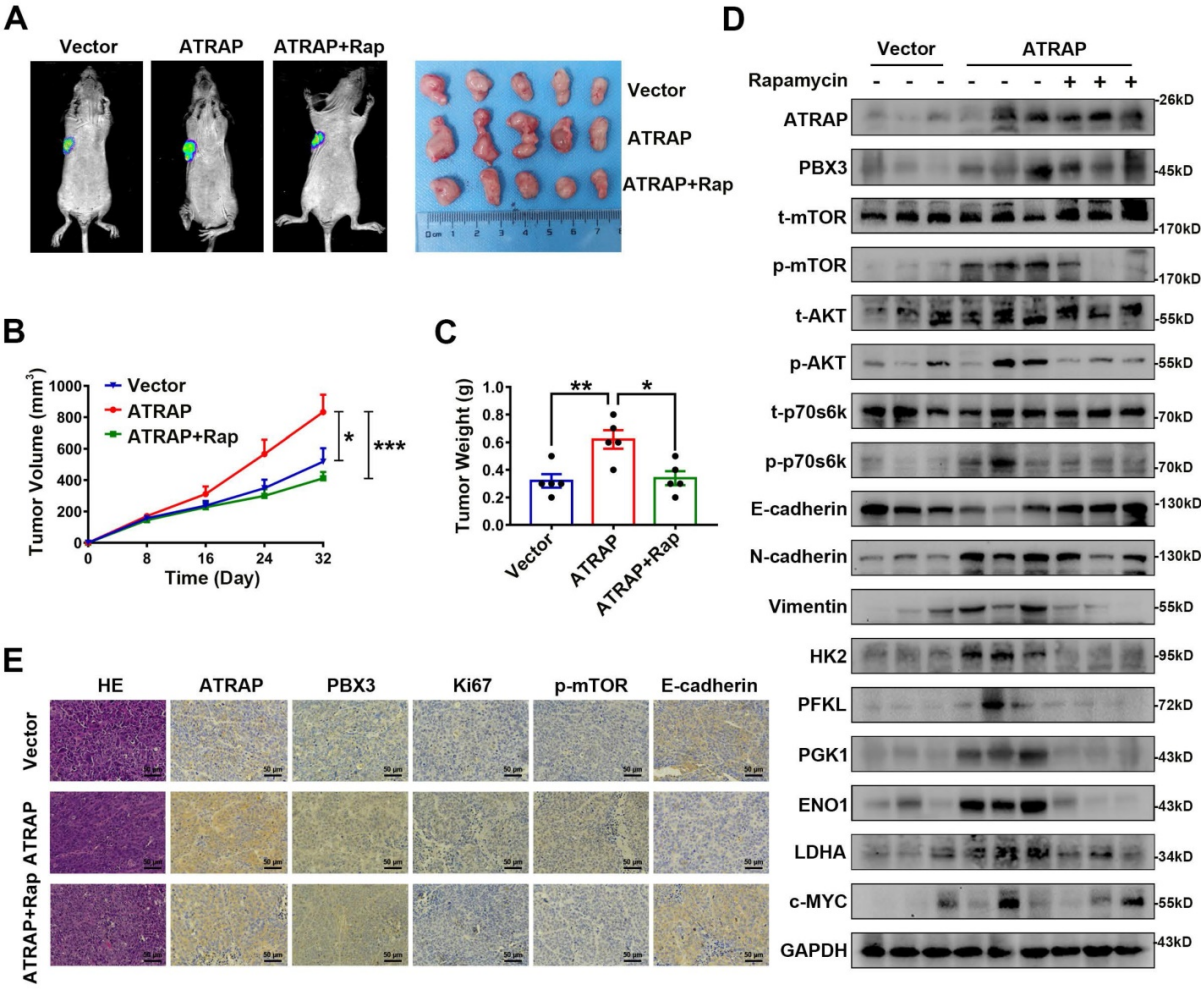
** Overexpression of ATRAP contributes to breast cancer progression *in vivo*.** T47D/vector cells and stable ATRAP-overexpression cells (the T47D cell type expressed luciferase) were subcutaneously injected into the armpit regions of the forelimb of nude mice. Mice receiving T47D/ATRAP xenografts were treated with or without rapamycin (2 mg/kg). **(A)** Tumor formation was monitored by bioluminescence imaging to assess the therapeutic effect of ATRAP overexpression, as well as rapamycin treatment and representative features of the tumors in the different treatment groups at 32 days. **(B)** The tumor volume in the nude mice from the three groups was measured at 8 days intervals from days 0 to 32 (n=5 mice in each group). **(C)** Tumor weight was measured in the different groups of mice. **(D)** Western blot analysis of the expression of the indicated markers in protein extracts obtained from harvested tumors. GAPDH served as a loading control. **(E)** Immunohistochemistry analysis to confirm the expression of ATRAP, PBX3, Ki67, p-mTOR, and E-cadherin in the indicated groups of tumor samples. Scale bar, 50 μm. Data represent mean ± SEM. Statistical significance was determined by ANOVA test (**B**) or two-tailed unpaired *t*-test (**C**). ^*^p < 0.05, ^**^p < 0.01, and ^***^p < 0.001.

To examine the biological effects of ATRAP on breast cancer progression *in vivo*, we employed a nude mouse xenograft model for breast cancer tumors. As shown in Figure 5A-C, tumors formed by ATRAP-overexpressing T47D cells were larger and heavier than the tumors formed by control cells. However, Rapamycin treatment resulted in significantly decreased volume and weight of tumors in these T47D ATRAP-overexpression xenograft groups. By contrast, the tumors formed by ATRAP-silenced UACC-812 cells were smaller and weighed less than those in the control group (Figure S7A-C). Moreover, compared with those of T47D control cells, the protein levels of activated AKT/mTOR pathway proteins, mesenchymal tissue markers, and glycolysis-related kinases were all significantly increased in tumors formed by T47D ATRAP-overexpression cells, whereas E-cadherin accumulation decreased, while rapamycin treatment reversed these effects (Figure 5D). Evaluation of tumors formed by UACC-812 ATRAP-knockdown cells indicated lower accumulation of activated AKT/mTOR pathway proteins, mesenchymal tissue markers, and glycolysis-related kinases, with elevated expression of E-cadherin compared with control cells (Figure S7D). These results were also confirmed by IHC imaging (Figure 5E and Figure S7E). Collectively, these findings strongly support our hypothesis that ATRAP performs an oncogenic role in breast cancer progression via activation of AKT/mTOR signaling *in vivo*.

### ATRAP is a transcriptional target of USF1

**Figure 6:**
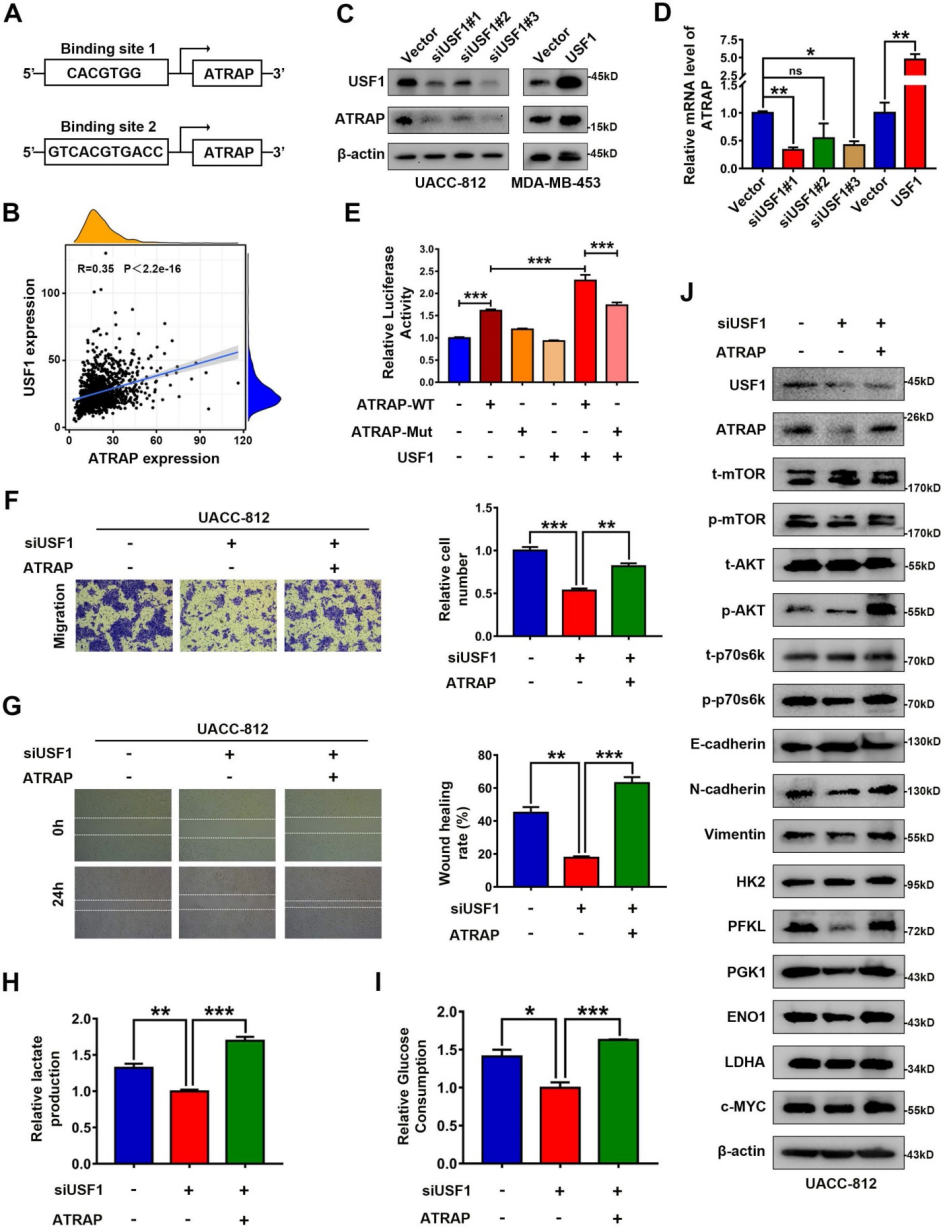
** USF1 upregulates ATRAP and induces breast cancer progression. (A)** Prediction of transcription factor binding site in the ATRAP promoter using the UCSC (http://genome.ucsc.edu) and JASPAR (http://jaspar.genereg.net/) databases. **(B)** The correlation between ATRAP and USF1 mRNA expression was identified by the TCGA database. **(C)** UACC-812 cells were transfected with USF1-siRNA (siUSF1#1, siUSF1#2, siUSF1#3) and MDA-MB-453 cells were transfected with USF1 overexpression plasmids. Cell lysates were subjected to immunoblot analysis with the indicated antibodies. **(D)** The mRNA levels of ATRAP were verified by qRT-PCR in indicated cells. **(E)** Relative luciferase activity in HEK-293T cells after the co-transfection of plasmid constructs (pcDNA3.1) containing the ATRAP promoter (WT and Mut) with or without a USF1 overexpressing construct. **(F, G)** Representative images (left panel) and quantification (right panel) of migratory cells analyzed with a transwell assay (**F**) and wound healing assay (**G**). **(H, I)** Relative lactate production level (**H**) and relative glucose consumption level (**I**) indicated that ATRAP overexpression reversed the inhibition of USF1 silencing in UACC-812 cells. **(J)** Proteins extracted from the indicated cells were subjected to western blotting to analyze the expression of ATRAP, USF1, p-mTOR, t-mTOR, p-AKT, t-AKT, p-p70s6k, t-p70s6k, EMT markers, and glycolysis enzymes. β-actin served as a loading control. Data represent mean ± SEM. Statistical significance was determined by Pearson's correlation analysis (**B**) or two-tailed unpaired *t*-test (**D-I**). ^*^p < 0.05, ^**^p < 0.01, and ^***^p < 0.001.

To identify the functional signatures of ATRAP-bound genes that are enriched in breast cancer cells, we analyzed the ATRAP promoter sequence using the UCSC (http://genome.ucsc.edu) and JASPAR (http://jaspar.genereg.net/) databases. These analyses identified USF1 as the most probable candidate for mediating the transactivation of ATRAP (Figure 6A and Figure S8A). In fact, a positive correlation between ATRAP and USF1 mRNA levels was observed in breast cancer samples from the TCGA database (Figure 6B). A growing body of evidence suggests that 1) USF1 is overexpressed in breast cancer cell lines and tissues; 2) USF1 can promote cell proliferation; and 3) USF1 expression is correlated with poor survival outcomes 33. Based on these findings, we re-examined the role of USF1 in breast cancer cells in publicly available datasets from TCGA. We found that USF1 expression was significantly upregulated in primary breast cancer tissues compared with that in normal breast tissue (Figure S8B). USF1 knockdown significantly inhibited the proliferation and migration of UACC-812 cells (Figure S8C-E). In addition, western blot analysis showed that siRNA-mediated suppression of USF1 inhibited activation of the AKT/mTOR pathway, as well as the occurrence of EMT, and decreased the levels of glycolytic kinases (Figure S8F). As expected, overexpression of USF1 remarkably increased both ATRAP protein and mRNA levels, while USF1 knockdown had the opposite effect on ATRAP at the protein and mRNA levels (Figure 6C and D). To explore how ATRAP mRNA is regulated by USF1, DNA fragments containing wild-type or mutant binding sequence are inserted into the promoter region of a firefly luciferase reporter plasmid. As we expected, luciferase activity from the reporter containing the wild-type binding sites is induced by ectopic expression of USF1 (Figure 6E).

In light of these findings that ATRAP was regulated by USF1, thereby contributing to breast cancer progression, we investigated the effects of USF1 on aggressive behavior in ATRAP-induced breast cancer. To this end, we transfected UACC-812 cells with a USF1-siRNA or empty vector and then co-transfected the USF1-siRNA knockdown line with pCMV3-ATRAP. Following western blot confirmation of USF1 and ATRAP expression (Figure 6J). We then conducted transwell and wound healing assays, and measured lactate production and glucose consumption by these cells. The results suggested that ATRAP is essential for USF1-mediated breast cancer progression (Figure 6F-I). In addition, western blots showed that USF1 knockdown significantly decreased the accumulation of activated AKT/mTOR proteins, reduced the occurrence of EMT, and decreased the levels of glycolysis-related kinases, while ATRAP overexpression abolished of the inhibitory effects of USF1 knockdown in these cells (Figure 6J). These findings showed that USF1 appears to directly bind to the ATRAP promoter to transactivate its expression.

### ATRAP is correlated with PBX3 and USF1 in breast cancer specimens and cells

**Figure 7:**
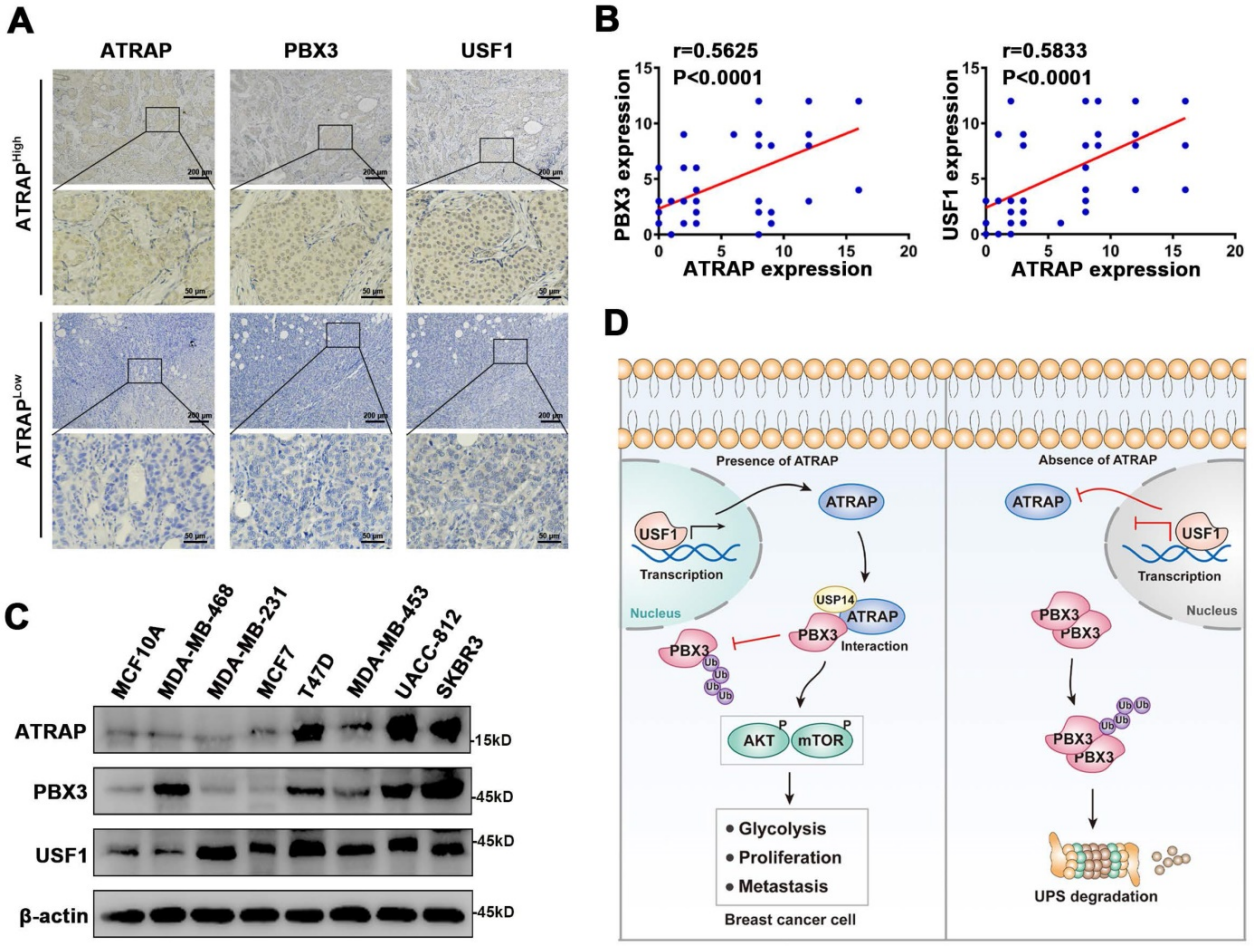
** Associations between ATRAP, PBX3, and USF1 in breast cancer tissues and cells. (A)** Representative images of immunohistochemical staining for ATRAP, PBX3, and USF1 in serial sections of breast cancer samples from patients with ATRAP-overexpression and ATRAP-low expression. Scale bar, 200 μm and 50 μm. **(B)** Correlation of ATRAP and PBX3 or USF1 staining intensities in clinical breast cancer tissues (n=49 patients).** (C)** ATRAP, USF1 and PBX3 protein expression levels in mammary epithelial cell lines (MCF10A) and 7 breast cancer cell lines were examined by western blot. **(D)** Schematic model of the role of ATRAP in breast cancer regulation. Data represent mean ± SEM. The *r* values and *p* values are from Pearson's correlation analysis (**B**).

To further examine the relationship between ATRAP and human breast cancer, we performed IHC staining of ATRAP, PBX3, and USF1 in 49 breast cancer patient specimens. Consistent with our observations, the distribution and intensity of ATRAP were both positively correlated with PBX3 and USF1 in breast cancer tissue specimens (Figure 7A and B). Subsequently, we examined the protein expression levels of USF1, ATRAP and PBX3 in breast epithelial cell (MCF10A) and 7 breast cancer cell lines. As shown in Figure 7C, USF1, ATRAP and PBX3 is detectable in all cell lines and higher than MCF10A cell. Besides, USF1 and PBX3 are highly expressed in several ATRAP-high cell lines, such as UACC-812, T47D and SKBR3. In contrast, MDA-MB-453 and MCF7 breast cancer cells exhibited relative low expression of USF1, ATRAP and PBX3. Therefore, these results further support that ATRAP expression is positively correlated with USF1 and PBX3 expression, which potentially leads to poor outcomes for patients with breast cancer.

## Discussion

Breast cancer patients commonly experience recurrence and metastasis after curative resection. For patients with aggressive tumors and multiple failed treatments, the prognoses are poor, and are at higher risk of dying from multiple organ failure. Thus, the development of novel effective therapies against breast cancer progression are urgently needed, which depends on clarification of its underlying molecular mechanisms. In the present study, we demonstrate that ATRAP expression is significantly increased in breast cancer tumor tissues and that overexpression of ATRAP is associated with a malignant breast cancer phenotype. Our findings indicate that, mechanistically, ATRAP deubiquitinates and stabilizes PBX3, leading to activation of the AKT/mTOR pathway and induction of cell EMT. Moreover, ATRAP is a downstream target gene of USF1, which functions as its transcriptional activator (Figure 7D). Furthermore, we found that in clinic, high levels of ATRAP combined with upregulation of USF1 and PBX3 can serve as a potentially reliable prognostic indicator for breast cancer patient outcomes.

The role of ATRAP's biological function in cancer has received little research attention. ATRAP, as a transmembrane protein, is not only expressed in the cell membrane but also in the cytoplasm 34-36. Its cellular localization in breast cancer has not been reported yet. Our results indicate that ATRAP localizes in the cytoplasm, which was consistent with the immunohistochemical results. We have demonstrated that the expression of ATRAP in breast cancer tissues is higher than that in normal tissues, and the expression level increases with the degree of malignancy. Importantly, ATRAP expressions were correlated with lymph node staging, and were an independent indicator for breast cancer patient prognoses. ATRAP was differentially expressed in breast cancer cell lines with different molecular types. We noted that ATRAP was low expressed in highly invasive triple-negative breast cancer cell lines. But there is no correlation between ATRAP and breast cancer molecular typing in large samples of breast cancer tissues. Therefore, it cannot be concluded that ATRAP has different effects on breast cancer cell lines of different molecular subtypes. The malignant phenotype of triple-negative breast cancer cell lines may also be driven by other genes, which reflects tumor heterogeneity and tumor biological behavior is a complex process of accumulation of multi-gene variants. Moreover, elevated ATRAP promotes cell proliferation, migration and invasion both *in vitro* and *in vivo*. Our research also revealed that ATRAP contributes to activation of the AKT/mTOR pathway, leading to tumor progression.

In addition, many oncogenes in the cancer catalog have reportedly acquired independence from growth signals 37. This independence from signal and transcription factors can lead to abnormal metabolism, which has been recognized as an emerging hallmark of cancer. Cancer cells frequently display increased aerobic glycolytic capacity 38. By providing energy to promote tumor cell growth, metastasis and chemoresistance. Therefore, exploration of dysregulated metabolism-related genes in cancer tissues will likely enhance our understanding of the regulatory mechanisms controlling breast cancer progression 22. AKT/mTOR signaling positively regulates the aerobic glycolysis involved in cancer cell proliferation and migration 39, 40. Herein, our data confirmed that ATRAP leads to both increased glucose consumption and lactate production. We also found ATRAP can regulate the expression of many glycolytic enzymes. Moreover, ATRAP facilitates aerobic glycolysis which in an AKT/mTOR pathway dependent manner. Previous study revealed that cancer cells can adapt to their malignant biological processes by increasing glucose uptake and lactate release 41, 22. Metabolic reprogramming has been shown to be a key feature of tumor progression, migration and angiogenesis 42. Therefore, ATRAP can improve glucose consumption and lactate production by activating the AKT/mTOR pathway, and providing energy to promote the growth and metastasis of breast cancer cells.

In our study, analysis conducted using the BioGRID database suggested that ATRAP can directly interact with PBX3, a pre-B-cell leukemia homeobox (PBX) transcription factor that is highly expressed in a variety of tumors and is associated with a poor prognosis for patients. In addition, PBX3 can promote cancer cell proliferation and invasion 30, 31, 43. It has also been shown to regulate a variety of cellular signaling pathways 44, 45 and can promote the occurrence of EMT 46, 47. Here, we demonstrate that knockdown of PBX3 significantly inhibits the proliferation and migration of UACC-812 cells. Subsequently, co-IP analysis confirmed the interaction between ATRAP and PBX3 protein. It is worth noting that PBX3 acts as a transcription factor, although immunohistochemical staining revealed a strong cytoplasmic PBX3 signal but little nuclear staining. Similarly, Lamprecht *at el*. 46 found that PBX3 is heterogeneously expressed in the cytoplasm of tumor cells, with its strongest expression at the tumor periphery in colorectal cancer. Ramberg *at el*. 31 also confirmed that PBX3 was increased in the cytosol of prostatic adenocarcinoma cells. Our study identified PBX3 as a target of ATRAP in breast cancer cells, and there is ample evidence that ATRAP binds to PBX3, protecting it from proteasomal degradation. PBX3 protein thus accumulates to high levels and its ubiquitination is significantly decreased in ATRAP overexpression cells.

Ubiquitination is involved in transcriptional regulation, DNA damage repair, cell cycle, cell apoptosis, vesicle transport, breast cancer stem cell maintenance and other physiological processes 48-53. The process of ubiquitination typically requires E1 ubiquitin-activating enzyme, E2 ubiquitin-conjugating enzymes, E3 ubiquitin-ligase enzymes, and the synergistic effects of deubiquitinating enzymes. Here, we show that ATRAP regulates AKT/mTOR signaling through PBX3 binding and stabilization. However, the detailed steps and participants in this regulatory process remain undefined. Future work will focus on identifying ubiquitinases or deubiquitinases that can directly interact with ATRAP during the regulation of PBX3 stability and ubiquitination. Mass spectrometry data showed that ATRAP can apparently interact with the deubiquitinating enzyme, USP14. Ubiquitin-specific protease 14 (USP14) is DUB family protein that reversibly associates with the 19S regulatory particle 54 and was shown to participate in the progression of multiple tumor types, including breast cancer 55-57. Specifically, USP14 can rescue proteins from degradation and serve as a quality control component by disassembling the ubiquitin chain from its substrate distal tip 58. In this study, we found that the regulation of PBX3 by ATRAP depends on USP14. Importantly, we further verified USP14 knockdown reduced ATRAP-induced PBX3 stabilization, which indicating that ATRAP stabilizes PBX3 via USP14 deubiquitination. We also showed that PBX3 participates in ATRAP-mediated breast cancer progression. These findings suggested that ATRAP directs USP14 deubiquitination PBX3 and further promotes cancer progression. However, the precise molecular mechanisms by which USP14 interacts with PBX3 in breast cancer remain unclear. Gaining a deeper understanding of the protein domain that USP14 binds to PBX3 would be interesting, which we aim to investigate in a future study and the results will be summarized into a separate manuscript to be reported in due course.

Given the strong effects related to dysregulation of ATRAP expression during breast cancer progression, we looked for transcription factors that could regulate ATRAP in order to define its complete regulatory interaction network. Analysis through the JASPAR database suggested that USF1 can activate ATRAP transcription. Upstream stimulating factor 1 (USF1) is a basic helix-loop-helix leucine zipper (bHLH-LZ) transcription factor that can combine with the E-box motif in the promoter regions of many genes, thereby functioning as a master regulator of several gene networks 59, 60. In this mechanism, USF1 recognition and binding results in the transcriptional activation of target genes involved in cell proliferation, invasion, and migration 61, 62. Other studies in mice have shown that USF1 and USF2 regulate ATRAP gene transcription through their interaction with an E-box in the ATRAP promoter, and also demonstrated functional E-box-USF1/USF2 binding in the human ATRAP promoter 63. Subsequently, we obtained results consistent with this literature by luciferase fusion reporter assays which confirmed that USF1 directly binds to ATRAP DNA sequence to transactivate its expression in breast cancer and that USF1 levels are positively correlated with ATRAP expression levels. Matsuda et al. 63 confirmed that USF1 suppresses ATRAP transcription, while USF2 activates it, and both interactions are mediated via binding to the ATRAP promoter E-box. But this effect was only observed in mDCT cells and in a unilateral ureteral obstruction (UUO) model in mice, and has not been confirmed in human patients or cell lines. In our experiments, we demonstrated that inhibition of USF1 expression can reduce breast cancer cell proliferation, movement, and invasion, while ATRAP overexpression can restore the inhibitory effects of USF1 on breast cancer cells.

Here, we showed that ATRAP activates the AKT/mTOR signaling and acts as an oncogene to promotes breast cancer progression. Moreover, we identified ATRAP can interact with PBX3 and stabilize it via USP14 deubiquitination. Rescue assay showed that ATRAP regulates breast cancer cell progression via PBX3. Previous study has reported that PBX3 can phosphorylate AKT and promote the proliferation and metastasis of tumors 44. Furthermore, AKT is phosphorylated at residues Thr308 and Ser473, and then triggers the activation of downstream targets (including mTOR, GSK-3, FOXOs and so on), which stimulates cell survival, proliferation, metabolism and drug resistance to promote tumor progression 64, 65. Consistent with this evidence, our study also indicated that PBX3 can activates AKT/mTOR pathway. For the upstream analysis, we found that USF1 directly binds to the ATRAP promoter to activate its expression, which further participated in tumor malignant transforming processes. Therefore, the USF1/ATRAP/PBX3 axis activates AKT/mTOR signaling and promotes breast cancer aggressiveness.

In conclusion, we uncovered a USF1-ATRAP-PBX3-AKT/mTOR axis that functions in breast cancer tumor progression both *in vitro* and *in vivo*. Importantly, ATRAP may be a useful prognostic indicator for breast cancer and could serve as a new potential therapeutic target. In future study, we expect to use transgenic mice to achieve precise regulation of gene expression and epigenetic modification *in vivo*, which will more accurately explain the biological role of ATRAP in breast cancer.

## Supplementary Material

Supplementary figures and tables 1-2.

Supplementary table 3.

Supplementary table 4.

Supplementary table 5.

## Figures and Tables

**Table 1:** Association of ATRAP level with clinical and pathological characteristics of breast cancer patients

Variables	Tumor ATRAP expression		*p* value
	ATRAP^Low^	ATRAP^High^	
**Age (year)**			0.572
≤40	26	39	
>40	107	190	
**Tumor size(cm)**			0.128
≤2	64	86	
>2; ≤5	64	130	
>5	5	13	
**Node stage**			0.025^*^
N0	80	105	
N1	21	62	
N2	20	32	
N3	12	30	
**Histological stage**			0.574
Level 1	9	13	
Level 2	95	171	
Level 3	8	9	
**Pathological types**			0.909
DCIS	7	12	
IDC	117	205	
ILC	1	2	
Special types	8	10	
**TNM stage**			0.115
I	40	47	
II	60	114	
III	33	68	
**Ki67**			0.603
≤14	17	25	
>14	92	170	
**ER**			1.000
-	42	73	
+	72	126	
**PR**			0.775
-	23	44	
+	91	154	
**HER2**			0.118
- ~ +	94	150	
++ ~ +++	19	49	
**P53**			0.896
-	75	137	
+	33	58	

Note: The Chi-square test was performed to analyze the relationship between ATRAP expression level and clinical pathological parameters. n=362. DCIS, Ductal carcinoma *in situ*; IDC, Invasive ductal carcinoma; ILC, Invasive lobular carcinoma. All *p*-values were two sided and the level of statistical significance was set at < 0.05.^ *^p < 0.05.

**Table 2:** Results of univariate and multivariate Cox proportional hazards analysis for disease-free survival (DFS) in breast cancer patients

Variables	Univariate Analysis			Multivariate Analysis		
	Hazard Ratio	95% Confidence Interval	*p*-value	Hazard Ratio	95% Confidence Interval	*p*-value
ATRAP staining (Low vs. High)	1.849	1.236-2.767	0.003^**^	1.636	1.009-2.651	0.046^*^
Age, year (≥40 vs. <40)	1.099	0.687-1.758	0.694			
Tumor size(cm) (>5 vs. >2; ≤5 vs. ≤2)	2.116	1.543-2.901	0.000^***^	1.447	0.979-2.140	0.064
Node stage (N0 vs. N1 vs. N2 vs. N3)	2.025	1.730-2.370	0.000^***^	1.764	1.446-2.152	0.000^***^
Histological stage (Level 1 vs. Level 2 vs. Level 3)	1.995	1.116-3.566	0.020^*^	1.892	0.911-3.931	0.088
Pathological types (DCIS vs. IDC vs. ILC vs. Special types)	1.015	0.732-1.408	0.930			
TNM stage (I vs. II vs. III)	2.901	2.193-3.837	0.000^***^			
Ki67 (≤14 vs. >14)	2.122	1.030-4.371	0.041^*^	1.338	0.640-2.798	0.439
ER (Negative vs. Positive)	0.738	0.497-1.094	0.131			
PR (Negative vs. Positive)	0.998	0.622-1.599	0.992			
HER2 (- ~ + vs. ++ ~ +++)	1.395	0.893-2.180	0.144			
P53 (Negative vs. Positive)	1.499	0.992-2.263	0.054			

Note: n=362. DFS, disease-free survival. DCIS, Ductal carcinoma *in situ*. IDC, Invasive ductal carcinoma. ILC, Invasive ductal carcinoma. *p* < 0.05 was regarded as statistically significant, *p* value was calculated using Cox proportional hazards regression. ^*^p < 0.05, ^**^p < 0.01, and ^***^p < 0.001.

## Abbreviations

ATRAP: Angiotensin II type 1 receptor-associated protein
PBX3: Pre-B cell leukemia homeobox 3
USP14: Ubiquitin-specific protease 14
USF1: Upstream stimulatory factor 1
mTOR: mammalian target of rapamycin
mTORC1: mTOR complex 1
mTORC2: mTOR complex 2
PI3K: phosphatidylinositol-3-kinase
TCGA: The Cancer Genome Atlas
IHC: Immunohistochemical
DFS: Disease-Free Survival
OS: Overall Survival
DEGs: Differentially Expressed Genes
CHX: Cycloheximide
DUBs: deubiquitinases
bHLH‐LZ: basic Helix‐Loop‐Helix Leucine Zipper
UUO: Unilateral Ureteral Obstruction
siRNAs: Small interfering RNAs
co-IP: co-Immunoprecipitation
