# Supplementary material for: USF1-ATRAP-PBX3 Axis Promote Breast Cancer Glycolysis and Malignant Phenotype by Activating AKT/mTOR Signaling
Source: Int J Biol Sci. 2022 Mar 14;18(6):2452–71. doi: 10.7150/ijbs.69134 (PMC8990462; doi:10.7150/ijbs.69134)
Supplement: Supplementary file 1 — Supplementary figures and tables 1-2. [file ijbsv18p2452s1.pdf]

Supplementary Information

USF1-ATRAP-PBX3 Axis Promote Breast Cancer Glycolysis And Malignant Phenotype By Activating AKT/mTOR Signaling

Dandan Wang et al.

Supplementary Tables

Table S1 Information of target sequences used in the study.

Oligonucleotides		
**siRNAs**	sense（5′-3′）	
siNC	5′-TTCTCCGAACGTGTCACGT-3′	
siPBX3#1	5′-GGAGGTTCTTCAGATAACT-3′	
siPBX3#2	5′-GGGTTTCAGGTCCTGAGAA-3′	
siPBX3#3	5′-GCCAAATTGACCCAGATCA-3′	
siUSF1#1	5′-GCTGGACAATGACGTGCTT-3′	
siUSF1#2	5′-GACGACTCGGGATGAGAAA-3′	
siUSF1#3	5′-CGCCGAGACAAGATCAACA-3′	
SiUSP14	5'-CTGGCA TATCGCTTACGTT-3'	
**shRNA**	sense（5′-3′）	antisense (5′-3′)
shATRAP#1	CCGGCGTAGTGCCTACCAGACGATTCTCGAGAATCGTCTGGTAGGCACTACGTTTTTG	AATTCAAAAACGTAGTGCCTACCAGACGATTCTCGAGAATCGTCTGGTAGGCACTACG
shATRAP#2	CCGGGCCATAAGCATGTTTCTGGGTCTCGAGACCCAGAAACATGCTTATGGCTTTTTG	AATTCAAAAAGCCATAAGCATGTTTCTGGGTCTCGAGACCCAGAAACATGCTTATGGC
shATRAP#3	CCGGCATTGTATTCTCAGGCTCCTACTCGAGTAGGAGCCTGAGAATACAATGTTTTTG	AATTCAAAAACATTGTATTCTCAGGCTCCTACTCGAGTAGGAGCCTGAGAATACAATG

Table S2 Information of antibodies used in the study.

**Antibodies**		
**REAGENT**	**Catalog and Source**	**Dilution**
ATRAP	Cat#134266; Absin	1:500
PBX3	Cat#12571-1-AP; Proteintech	1:1000
USF1	Cat#sc-390027; Santa Cruz	1:500
USP14	Cat#14517-1-AP; Proteintech	1:1000
E-cadherin	Cat#3195; Cell Signaling Technology	1:1000
N-cadherin	Cat#13116; Cell Signaling Technology	1:1000
Vimentin	Cat#10366-1-AP; Proteintech	1:1000
t-mTOR	Cat#2983; Cell Signaling Technology	1:1000
p-mTOR	Cat#5536; Cell Signaling Technology	1:1000
t-AKT	Cat#4685; Cell Signaling Technology	1:1000
P-AKT	Cat#9271; Cell Signaling Technology	1:1000
t-p70s6k	Cat#2708; Cell Signaling Technology	1:1000
p-p70s6k	Cat#9234; Cell Signaling Technology	1:1000
HK2	Cat#22029-1-AP; Proteintech	1:2000
PFKL	Cat#ab181064; Abcam	1:5000
PGK1	Cat#17811-1-AP; Proteintech	1:1000
ENO1	Cat#11204-1-AP; Proteintech	1:1000
PKM2	Cat# AF5234; Affinity	1:1000
LDHA	Cat#19987-1-AP; Proteintech	1:1000
c-MYC	Cat#10828-1-AP; Proteintech	1:1000
Flag	Cat#20543-1-AP; Proteintech	1:1000
β-actin	Cat#TA-09; ZSGB-BIO	1:1000
GAPDH	Cat#60004-1-Ig; Proteintech	1:50000

Supplementary Figures

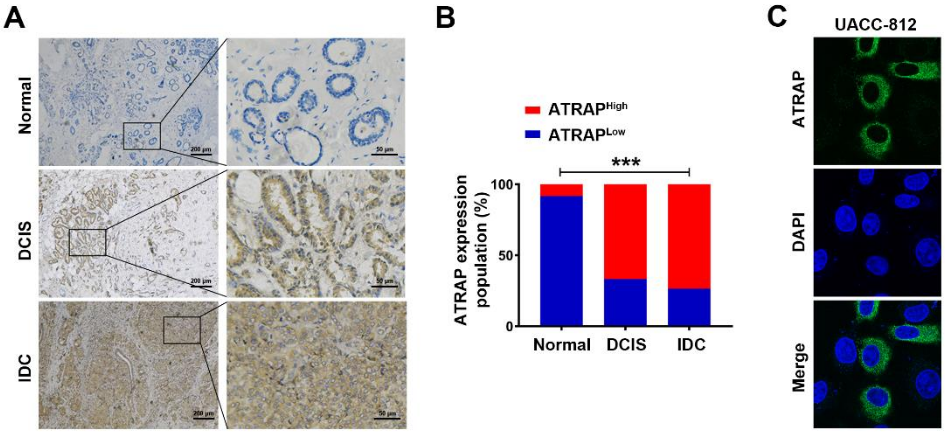

Figure S1. ATRAP expression in breast cancer tissues and cell. (A) Immunohistochemical detection of ATRAP expression in normal breast tissue, ductal carcinoma in situ and invasive ductal carcinoma. Scale bar, 200 μm and 50 μm. **(B)** The proportion of ATRAP expression levels in normal breast tissue, ductal carcinoma in situ and invasive ductal carcinoma. **(C)** The cellular localization of ATRAP in UACC-812 cell. $^{***}p < 0.001$.

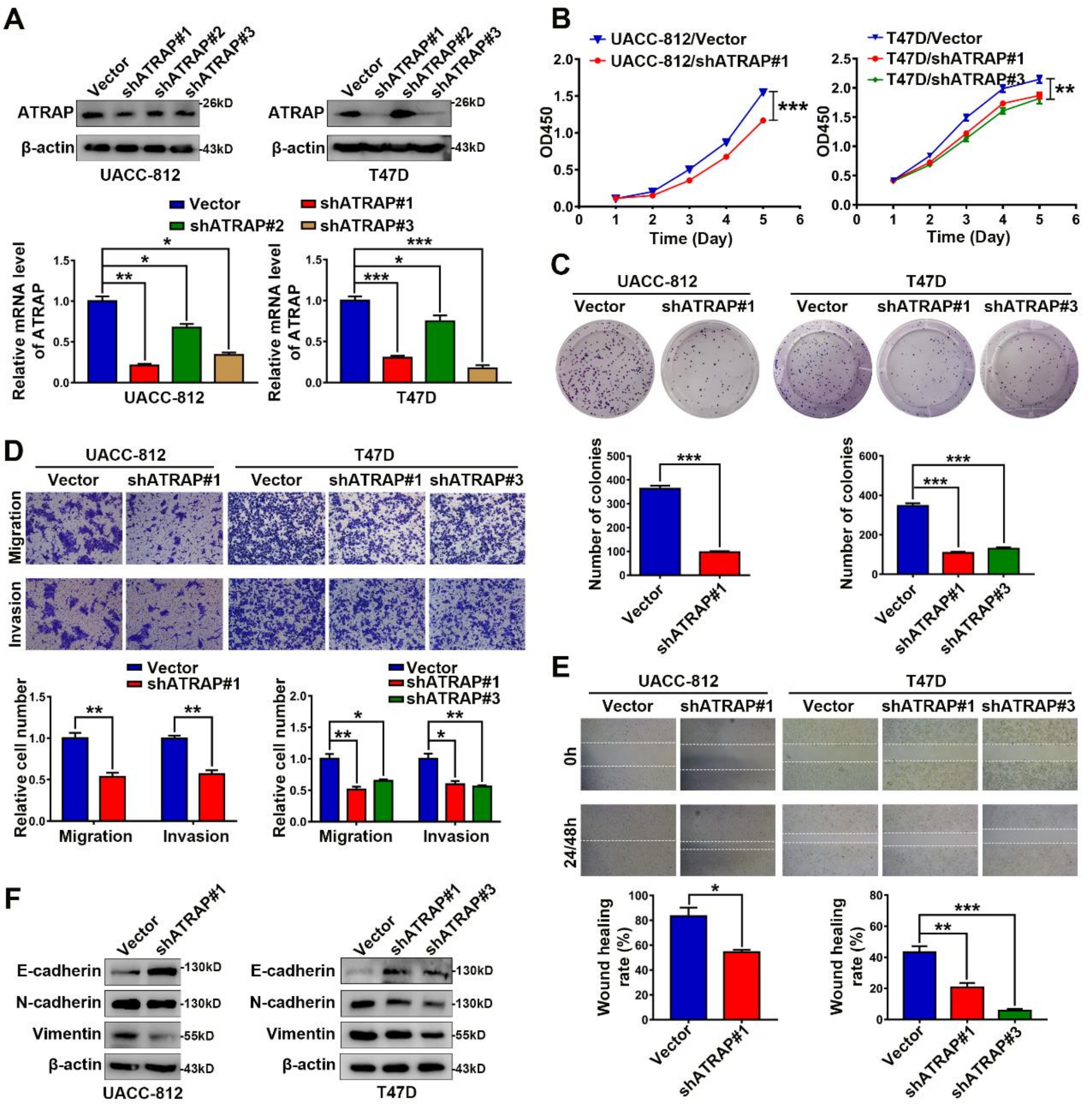

Figure S2. Depletion of ATRAP dramatically inhibits cell motility *in vitro*. (A) ATRAP protein and mRNA expression levels were determined by western blotting and qRT-PCR in UACC-812 (left panel) and T47D (right panel) cells stably expressing empty vector or shATRAP (shATRAP1#1, 2, 3). **(B)** The viability of UACC-812/shATRAP#1 (left panel) and T47D/shATRAP#1 and #3 (right panel), and corresponding vector control cells were analyzed by a CCK-8 assay. **(C)** The colony-forming efficiency of UACC-812/shATRAP#1, T47D/shATRAP#1 and #3, and corresponding vector control cells was determined. **(D)** Transwell assays were conducted to assess cell migration and invasion after ATRAP knockdown in UACC-812 and T47D cells compared with corresponding

vector control cells. **(E)** A wound healing assay was performed in the indicated cells as described in D. **(F)** Protein expression levels of E-cadherin, N-cadherin and vimentin were analyzed by western blotting. $^{*}p < 0.05$, $^{**}p < 0.01$, and $^{***}p < 0.001$.

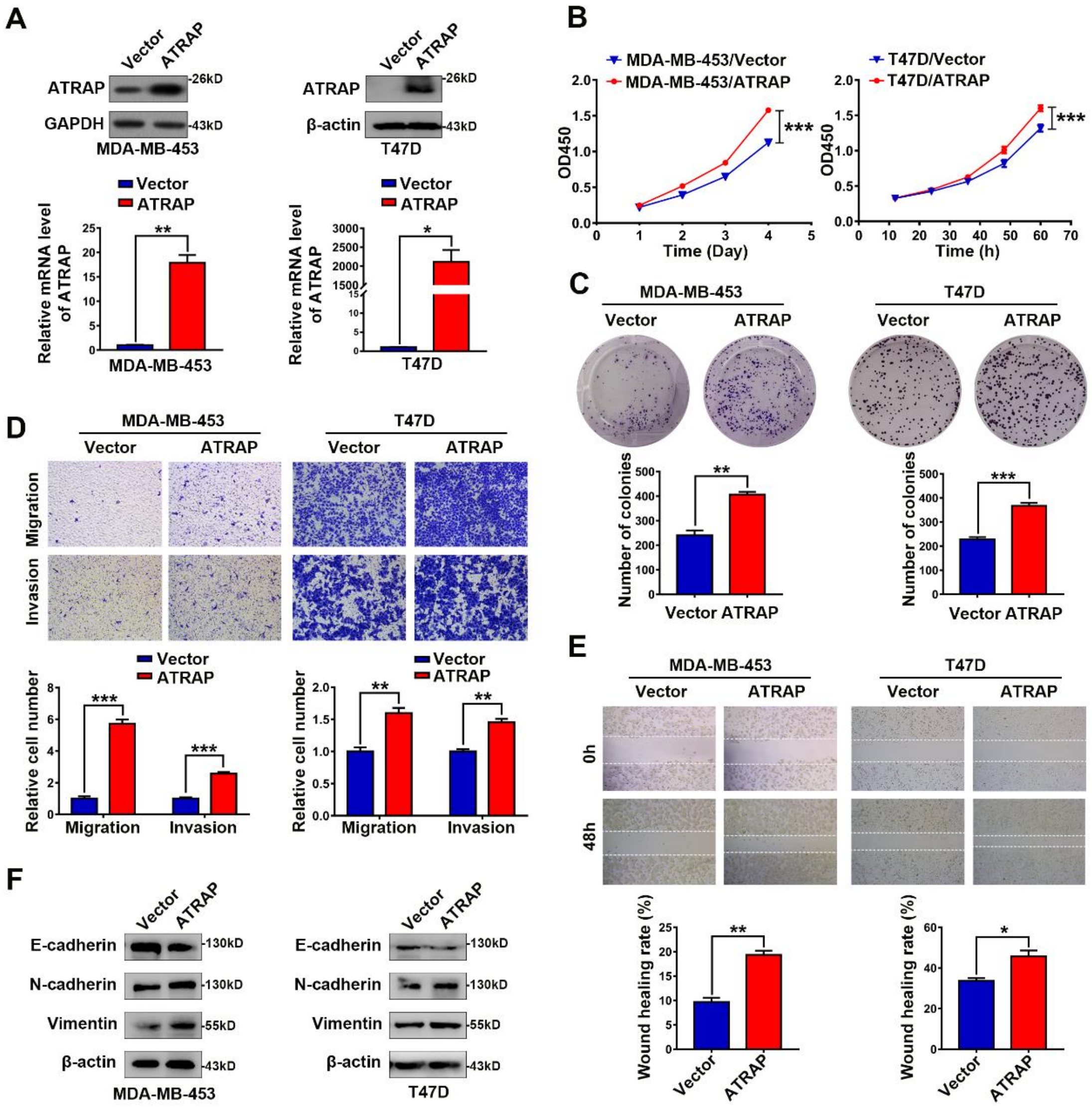

Figure S3. Upregulation of ATRAP promotes malignant behavior of breast cancer *in vitro*. (A) ATRAP protein and mRNA expression levels were determined by western blotting and qRT-PCR in MDA-MB-453 (left panel) stably expressing empty vector or ATRAP and T47D (right panel) transfected PCMV3-Flag-ATRAP cells. **(B)** Cell proliferation in vector control and ATRAP

overexpressing cells was detected with CCK-8 assays. **(C)** Colony formation assays in expressing vector control and ATRAP cells. **(D)** Transwell assays were conducted to assess cell migration and invasion after ATRAP overexpression in MDA-MB-453 and T47D cells compared with corresponding vector control cells. **(E)** A wound healing assay was performed in the indicated cells as described in D. **(F)** Protein expression levels of E-cadherin, N-cadherin and vimentin were analyzed by western blotting. $^{*}p < 0.05$, $^{**}p < 0.01$, and $^{***}p < 0.001$.

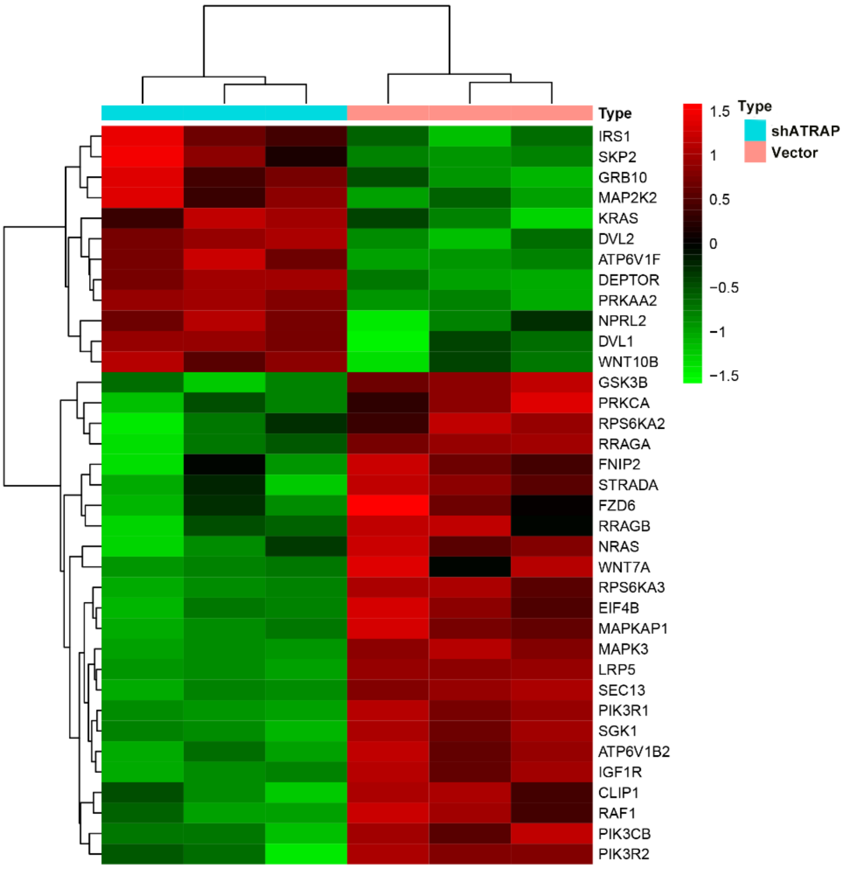

Figure S4. Correlation between ATRAP and mTOR signaling pathway. Heatmaps showing that ATRAP was significantly correlated with mTOR signaling pathway in control and ATRAP-knockdown breast cancer cells. Red and green indicate high and low mRNA expression levels,

respectively.

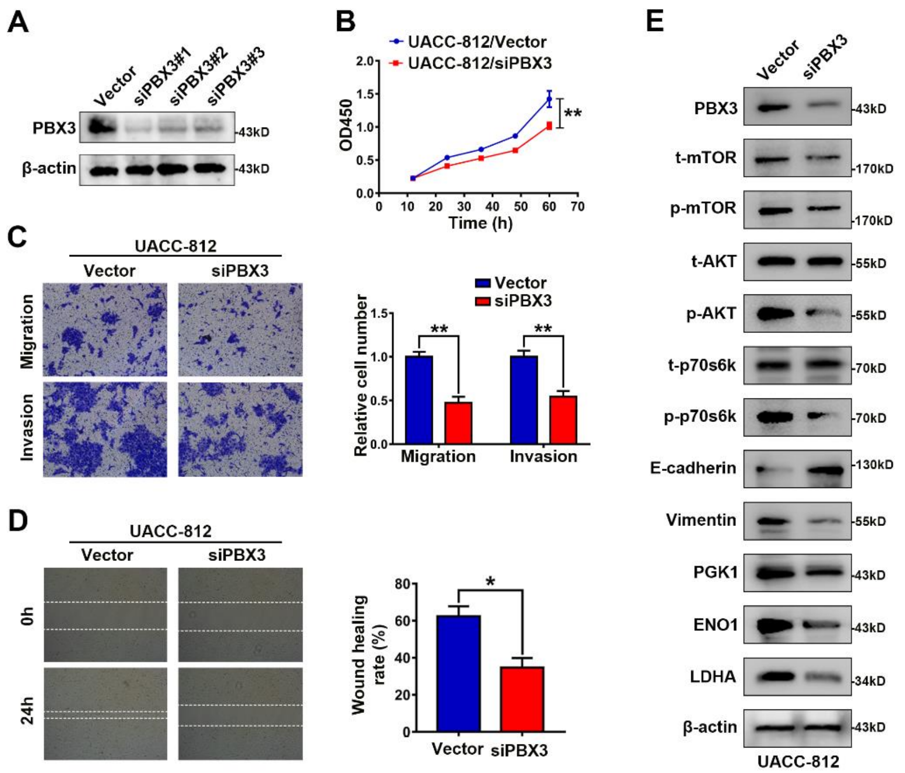

Figure S5. PBX3 promotes breast cancer cell proliferation and motility. (A) PBX3 protein expression levels were determined by western blotting in UACC-812 cells expressing empty vector or siPBX3 (siPBX3#1, 2, 3). **(B)** Viability analyzed by a CCK-8 assay in UACC-812 cells after knockdown of PBX3 with siRNA (siPBX3#1). **(C)** Transwell assays were conducted to assess cell migration and invasion in UACC-812/siPBX3#1 cells compared with corresponding vector control cells. **(D)** A wound healing assay was performed in the indicated cells. **(E)** Analysis of the expression of indicated protein markers in PBX3 knockdown cells by western blotting. $^{*}p < 0.05$, and $^{**}p < 0.01$.

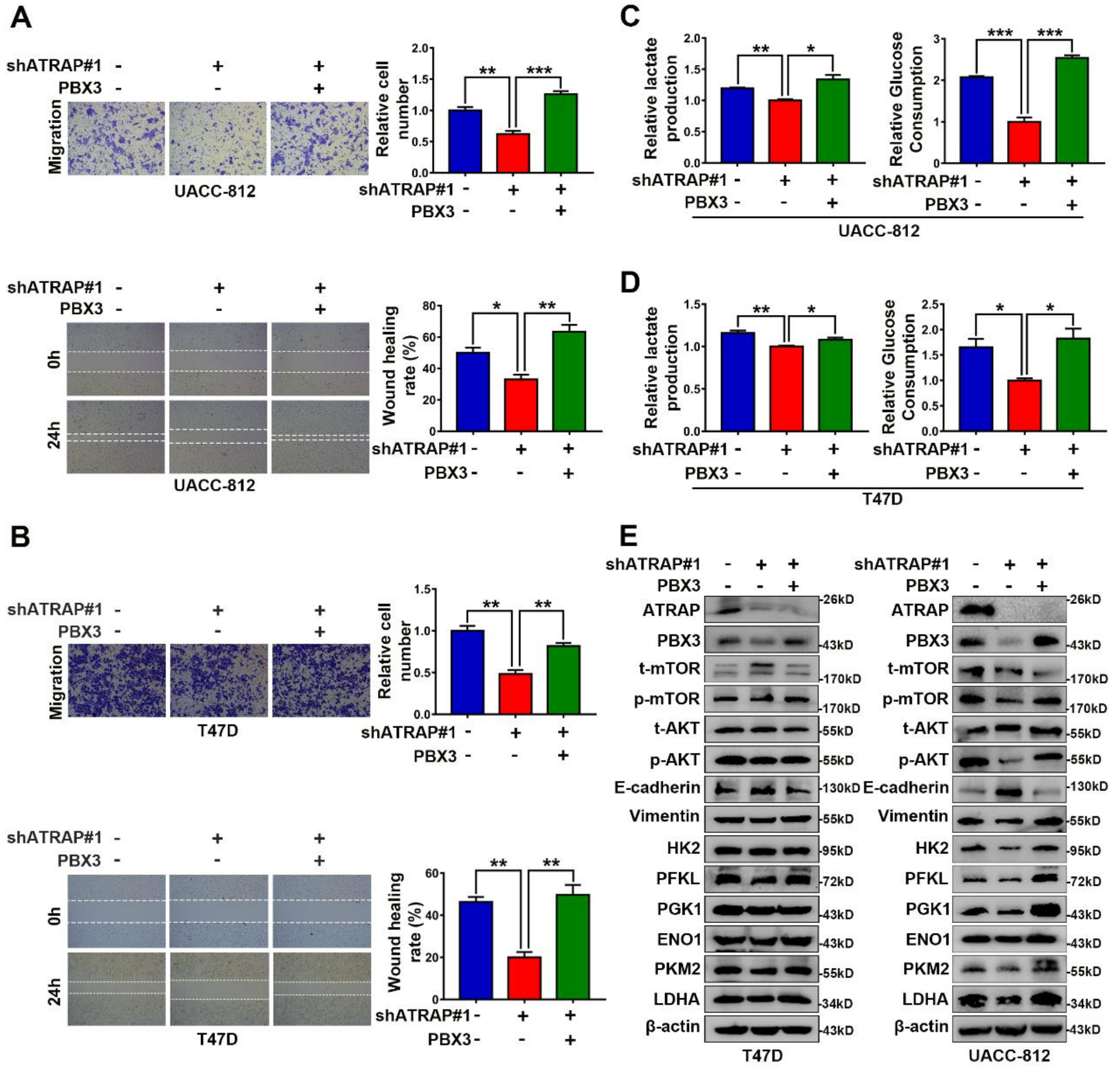

Figure S6. PBX3 is required for ATRAP-mediated malignant behavior of breast cancer. (A) Rescue assays for transwell (upper panel) and wound healing (lower panel) assays were performed after PBX3 overexpression in UACC-812 cells stably silencing ATRAP. **(B)** Rescue assays for transwell (upper panel) and wound healing (lower panel) assays were performed after PBX3 overexpression in T47D cells stably silencing ATRAP. **(C)** Rescue assays for relative lactate production level (left panel) and relative glucose consumption level (right panel) were analyzed after PBX3 overexpression in UACC-812 cells stably silencing ATRAP. **(D)** Rescue assays for relative lactate production level (left panel) and relative glucose consumption level (right panel) were analyzed after PBX3 overexpression in T47D cells stably silencing ATRAP. **(E)** The indicated

cell lysates were extracted to analyze the expression of indicated markers by western blot. $^{*}p < 0.05$, $^{**}p < 0.01$, and $^{***}p < 0.001$.

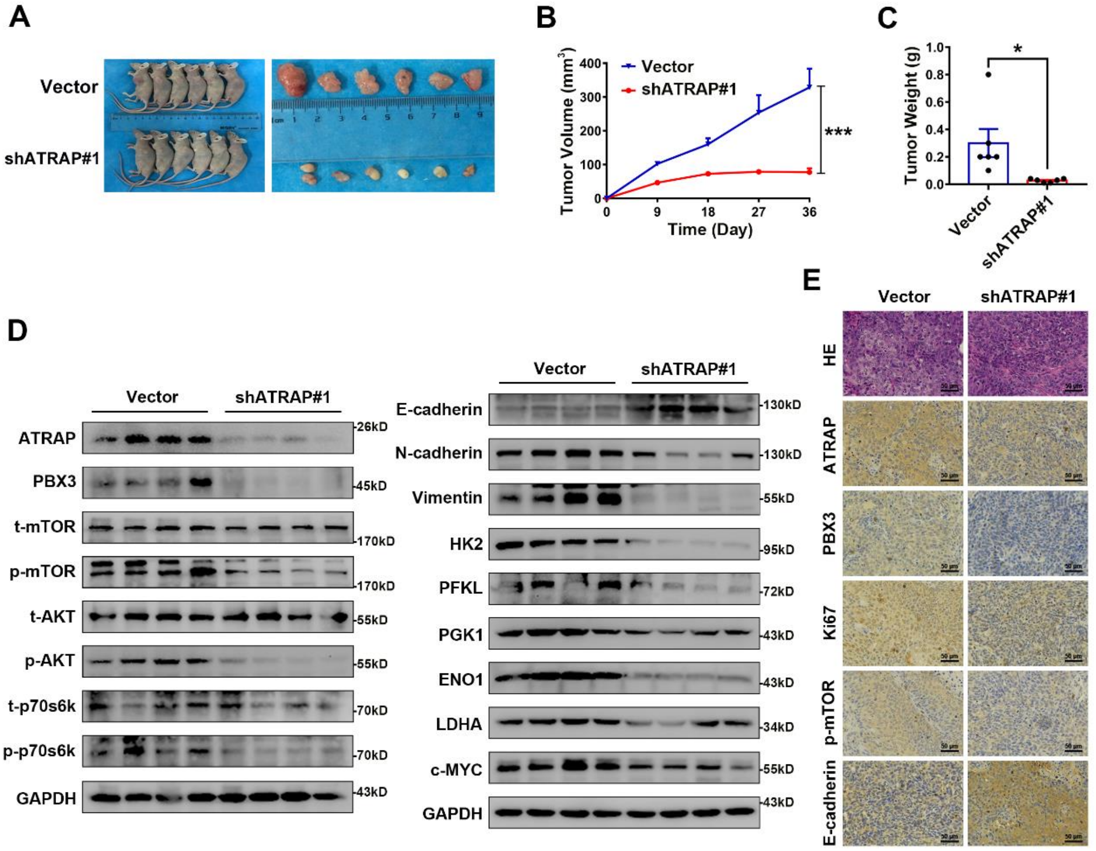

Figure S7. Silencing ATRAP suppresses breast cancer progression *in vivo*. UACC-812/vector cells and stable ATRAP-knockdown (shATRAP) cells were subcutaneously injected into the left armpit regions of the forelimb of nude mice. **(A)** Analysis of representative features of the tumors in the different groups at 36 days to assess the therapeutic effect of ATRAP knockdown. **(B)** The tumor volume in the nude mice from UACC-812/vector and UACC-812/shATRAP groups was measured at 9 days intervals from days 0 to 36 (n=6 mice in each group). **(C)** Tumor weight was measured in the different groups of mice. **(D)** Western blot analysis of the expression of the indicated markers in protein extracts obtained from harvested tumors. **(E)** Immunohistochemistry analysis to

confirm expression of ATRAP, PBX3, Ki67, p-mTOR, and E-cadherin in the indicated groups of tumor samples. Scale bar, 50 µm. $^{*}p < 0.05$, and $^{***}p < 0.001$.

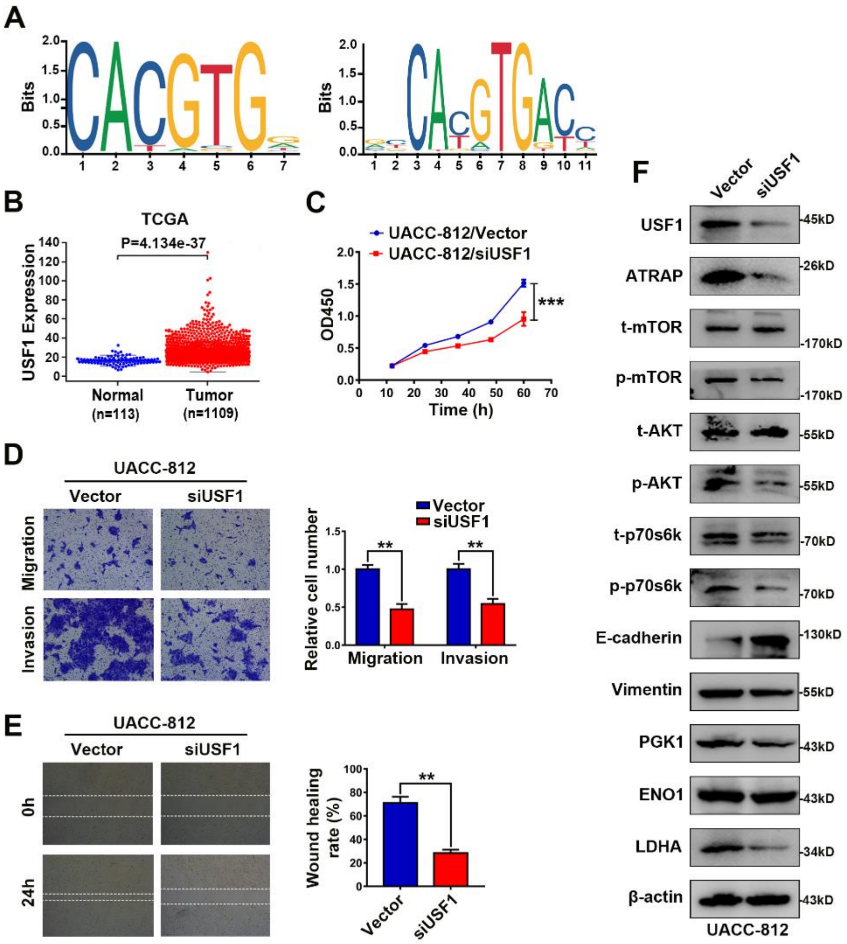

Figure S8. USF1 as an oncogene in breast cancer. (A) Prediction of transcription factor binding site in the ATRAP promoter using the UCSC (http://genome.ucsc.edu) and JASPAR (http://jaspar.genereg.net/) databases. **(B)** Expression profiles of USF1 mRNA in primary breast cancer tissues (n=1,109) and normal breast tissues (n=113; p<0.001) in the TCGA database. **(C)** Viability analyzed by a CCK-8 assay in UACC-812 cells after knockdown of USF1 with siRNA (siUSF1). **(D)** Transwell assays were conducted to assess cell migration and invasion in UACC-812/siUSF1 cells compared with corresponding vector control cells. **(E)** A wound healing assay was

performed in the indicated cells. **(F)** Analysis of the expression of indicated protein markers in USF1 knockdown cells by western blotting. $^{**}p < 0.01$, and $^{***}p < 0.001$.
